# ALS- and FTD-associated missense mutations in TBK1 differentially disrupt mitophagy

**DOI:** 10.1073/pnas.2025053118

**Published:** 2021-06-07

**Authors:** Olivia Harding, Chantell S. Evans, Junqiang Ye, Jonah Cheung, Tom Maniatis, Erika L. F. Holzbaur

**Affiliations:** ^a^Department of Physiology, Perelman School of Medicine, University of Pennsylvania, Philadelphia, PA 19104;; ^b^Aligning Science Across Parkinson’s Collaborative Research Network, Chevy Chase, MD 20815;; ^c^Department of Biochemistry and Molecular Biophysics, Vagelos College of Physicians and Surgeons, Columbia University, New York, NY 10032;; ^d^Zuckerman Mind Brain and Behavior Institute, Columbia University, New York, NY 10027;; ^e^Special Projects Group, New York Structural Biology Center, New York, NY 10027;; ^f^New York Genome Center, New York, NY 10013

**Keywords:** mitophagy, OPTN, TBK1, Parkin, neurodegeneration

## Abstract

TANK-binding kinase 1 (TBK1) is a multifunctional kinase with an essential role in mitophagy, the selective clearance of damaged mitochondria. More than 90 distinct mutations in TBK1 are linked to amyotrophic lateral sclerosis (ALS) and fronto-temporal dementia, including missense mutations that disrupt the abilities of TBK1 to dimerize, associate with the mitophagy receptor optineurin (OPTN), autoactivate, or catalyze phosphorylation. We investigated how ALS-associated mutations in TBK1 affect Parkin-dependent mitophagy using imaging to dissect the molecular mechanisms involved in clearing damaged mitochondria. Some mutations cause severe dysregulation of the pathway, while others induce limited disruption. Mutations that abolish either TBK1 dimerization or kinase activity were insufficient to fully inhibit mitophagy, while mutations that reduced both dimerization and kinase activity were more disruptive. Ultimately, both TBK1 recruitment and OPTN phosphorylation at S177 are necessary for engulfment of damaged mitochondra by autophagosomal membranes. Surprisingly, we find that ULK1 activity contributes to the phosphorylation of OPTN in the presence of either wild-type or kinase-inactive TBK1. In primary neurons, TBK1 mutants induce mitochondrial stress under basal conditions; network stress is exacerbated with further mitochondrial insult. Our study further refines the model for TBK1 function in mitophagy, demonstrating that some ALS-linked mutations likely contribute to disease pathogenesis by inducing mitochondrial stress or inhibiting mitophagic flux. Other TBK1 mutations exhibited much less impact on mitophagy in our assays, suggesting that cell-type–specific effects, cumulative damage, or alternative TBK1-dependent pathways such as innate immunity and inflammation also factor into the development of ALS in affected individuals.

## Significance

Missense mutations in TANK-binding kinase 1 (TBK1) have diverse biophysical and biochemical effects on the molecule and are associated with the neurodegenerative diseases amyotrophic lateral sclerosis (ALS) and fronto-temporal dementia. TBK1 plays an essential role in clearing damaged mitochondria. Here, we investigate the impact of 10 ALS-linked TBK1 mutations on the critical early stage of mitophagy. We find that both TBK1 recruitment and kinase activity contribute to the clearance of the damaged mitochondria. Furthermore, in neurons, expression of TBK1 mutants alone affects mitochondrial network health. Our investigation utilizes disease-linked mutations to further refine the current model of mitophagy, identifying regulatory crosstalk between the kinases TBK1 and ULK1, and providing insights into the roles of TBK1 in neurodegenerative pathogenesis.

TNF receptor–associated family member–associated NF-κB activator (TANK)-binding kinase 1 (TBK1) plays a critical role in several cellular pathways implicated in the neurodegenerative disease amyotrophic lateral sclerosis (ALS), including selective clearance of mitochondria and regulation of inflammation. More than 90 mutations in TBK1 have been linked to ALS, including several mutations identified in patients with the co-occurring degenerative disease, fronto-temporal dementia (ALS-FTD) (1, 2). Some TBK1 mutations are classified as loss of function variants while others are missense mutations with unclear contributions to disease pathogenesis (1, 3–6). The latter category includes mutations shown to disrupt the ability of TBK1 to dimerize, associate with the mitophagy receptor optineurin (OPTN), autoactivate, or catalyze phosphorylation (7–9). Given the importance of TBK1 in mitophagy (10), and the necessity of mitochondrial quality control to the maintenance of neuronal homeostasis (11, 12), functional analysis of ALS-associated missense mutations in TBK1 is necessary to determine the impact of mutant TBK1 in the neurodegeneration characteristic of ALS.

**Fig. 1.**
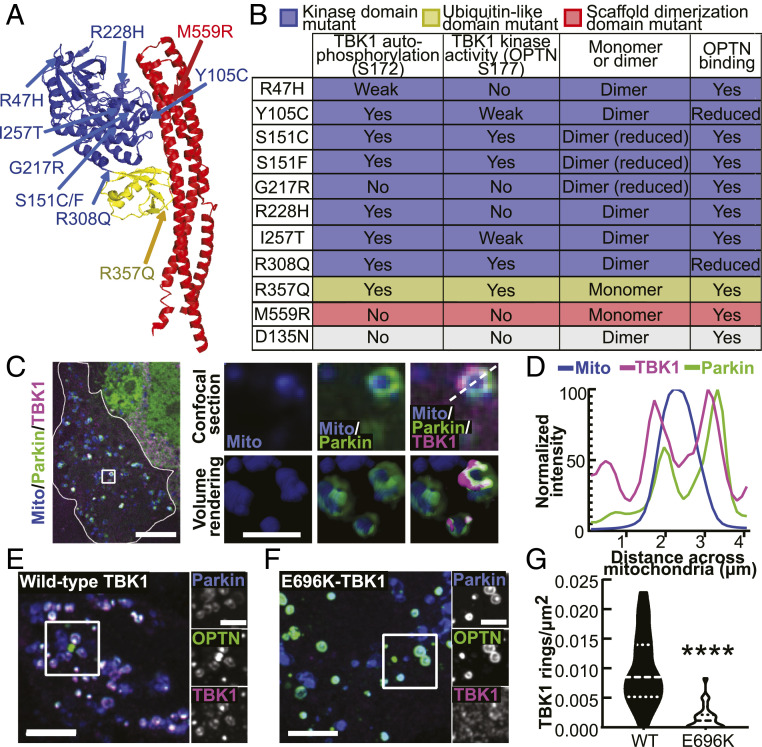
ALS-linked TBK1 mutations are found throughout the molecule and induce biochemical, biophysical, and cellular deficits. (*A*) Protein databank structure for TANK-binding kinase 1 (TBK1) (PDB 4IWO) (13). Domains are designated by color coding: kinase domain residues 1 to 308 (blue), ubiquitin-like domain residues 309 to 387 (yellow), and scaffolding dimerization domain residues 388 to 657 (red). ALS-linked mutations are indicated by arrows and labels of their respective colors. Some mutations likely disrupt the structure of TBK1, a phenomenon not represented by this model. (*B*) Table summarizing biochemical results for the ALS-linked mutants published by Ye et al. (8) and the engineered kinase-inactive D135N-TBK1 (gray). (*C*) Confocal section of a HeLa cell (outlined in white) expressing a mitochondria-localized fluorophore (blue), Parkin (green), and WT-TBK1 (magenta), fixed after treatment with CCCP for 90 min. The *Inset* (white box) and zoom images (*Right*) exhibit rounded mitochondria that have recruited Parkin and TBK1. A volume rendering is also shown (*Right*, bottom row). (Scale bars: zoom out, 10 μm; zoom in, 2 μm.) (*D*) Relative signal intensities for mitochondria, Parkin, and TBK1 are quantified across the diameter of a damaged mitochondria (white dashed line in *C*, zoom). (*E* and *F*) HeLa cells depleted of endogenous TBK1 expressing Parkin, OPTN, and WT- (*E*) or E696K- (*F*) TBK1, fixed after treatment with CCCP for 90 min. *Inset* (white box) and zoom images (*Left*) demonstrate multiple rings with colocalized mitophagy components. (Scale bars: zoom out, 10 μm; zoom in, 4 μm.) (*G*) Quantification of *E* and *F* as rings/μm^2^ for each cell. *n* = 22 to 25 cells from three independent experiments. Dashed line, median; dotted lines, 25th and 75th quartiles. ****P < 0.0001 by Student’s unpaired *t* test. Images *E* and *F* shown here are insets; for representative images of whole fields, reference *SI Appendix*, Fig. S2*B*.

TBK1 has three primary domains, 1) a kinase domain, 2) a ubiquitin-like domain, and 3) a scaffold dimerization domain, which are followed by a flexible C terminus domain (CTD) (Fig. 1*A*) (13–15). Two TBK1 monomers dimerize along their scaffold dimerization domains, while kinase activity is activated via autophosphorylation of the critical serine residue 172 (S172) within the activation loop of the kinase domain (14). Due to the conformation of the TBK1 dimer, it is unlikely that the monomers within a dimer can self-activate, so multimer formation is thought to be required for trans-autophosphorylation and kinase activation (13, 14). TBK1 multimerization may be promoted by association of TBK1 via its CTD with adaptor proteins including OPTN, TANK, Sintbad, and NAK-associated protein 1 (NAP1) (7, 16, 17). ALS-linked missense mutations are distributed throughout the protein, with some mutations disrupting dimerization, kinase activity, or both and others disrupting the association of TBK1 with adaptors, potentially inhibiting TBK1 multimerization and activation (Fig. 1*B*) (3, 6–8).

TBK1 is an essential regulator of mitophagy, a stepwise pathway for clearance of damaged mitochondria (10, 18). Mitophagy is triggered by loss of mitochondrial membrane potential, leading to the stabilization of PTEN-induced putative kinase 1 (PINK1) on the outer mitochondrial membrane (OMM) (19) where it phosphorylates ubiquitin (20). Phosphorylated ubiquitin recruits the E3 ubiquitin ligase, Parkin (20, 21), which is activated by PINK1 phosphorylation and then ubiquitinates OMM proteins (20, 22–25). These modifications promote proteasomal degradation of mitofusins, preventing the damaged organelle from re-fusing with the healthy mitochondrial network and resulting in a small, rounded mitochondrion (26). Ubiquitination of OMM proteins also promotes recruitment of the mitophagy receptors OPTN, nuclear dot 52 kDa protein (NDP52), Tax1-binding protein 1 (TAX1BP1), next to BRCA gene 1 protein (NBR1), and p62/sequestosome1 (10, 27–30), though OPTN and NDP52 are sufficient and redundant in carrying out mitochondrial clearance in HeLa cells (29). Phosphorylation of OPTN at S177 by TBK1 at the OMM enhances the binding of OPTN to ubiquitin chains (18). OPTN then drives recruitment of the core autophagy machinery, including the unc-51-like autophagy activating kinase (ULK1) complex, to initiate formation of the double membraned phagophore that engulfs the damaged organelle (31–33). In this process, microtubule-associated protein 1A/1B-light chain 3 (LC3) is lipidated and subsequently incorporated into the elongating phagophore (10, 27, 34). The LC3-interacting region of OPTN facilitates efficient engulfment by the autophagosome (10), while TBK1-mediated phosphorylation of OPTN enhances the binding of the receptor to LC3 (35). A feed-forward mechanism in which initial LC3-positive membranes recruit more OPTN and NDP52 leads to accelerated mitochondrial engulfment (36). The newly formed compartment fuses with lysosomes to complete degradation of the organelle (30, 37, 38).

We undertook a functional analysis of ALS-associated TBK1 missense mutations that have been characterized by biochemical and biophysical assays but confer unknown effects on the cellular pathways that involve TBK1. We determined the extent of recruitment of TBK1 mutants to depolarized, Parkin-positive mitochondria, the effect of mutant TBK1 expression on OPTN recruitment and phosphorylation, and the resulting downstream engulfment of fragmented mitochondria by LC3-positive autophagosomes. Expression of some ALS-linked mutations profoundly disrupted TBK1 recruitment and activity during mitochondrial clearance, while others only marginally affected the pathway. Neurons expressing TBK1 mutations demonstrated higher baseline levels of mitochondrial stress and an inability to manage induced oxidative damage, both of which may contribute to neurodegeneration. Our data suggest a nuanced model of TBK1 function, wherein TBK1 phosphorylates OPTN directly, while TBK1 recruitment also facilitates OPTN phosphorylation via an ULK1-dependent pathway. Furthermore, we demonstrate that ALS and ALS-FTD–associated missense mutations in TBK1 can lead to disordered or delayed mitochondrial clearance and a cellular deficiency in mitochondrial homeostasis.

## Results

In order to test whether mutations in TBK1 affect mitophagy, we used a well-characterized assay in HeLa-M cells, in which mitochondria were depolarized with the mitochondrial membrane disrupter, carbonyl-cyanide *m*-chlorophenyl-hydrazone (CCCP), and components of the mitophagy pathway were visualized by fluorescent microcopy (10, 27). We depleted cells of endogenous TBK1 and expressed SNAP- or Halo-tagged TBK1 (*SI Appendix*, Fig. S1 *A* and *B*) along with a fluorescently tagged mitochondrial marker, Parkin, OPTN, or LC3. While some constructs had a lower transfection efficiency as compared to wild-type (WT)-TBK1, most were expressed at similar cellular levels, with the exceptions of S151F and M559R, which exhibited slightly but statistically higher cellular expression (*SI Appendix*, Fig. S1 *C* and *D*). Under basal conditions, TBK1 was mostly cytosolic with intermittent puncta that did not associate with Parkin (*SI Appendix*, Fig. S1*E*).

With 90 min of CCCP treatment, Parkin, OPTN, TBK1, and LC3 assembled in a molecular platform at the OMM that appears as a ring surrounding a rounded mitochondrion in single-plane confocal sections (10); in Z-stacks, the complete engulfment of the mitochondrion is apparent (Fig. 1 *C* and *D* and *SI Appendix*, Fig. S2 *A* and *B*) (30). The time course of ring formation observed in HeLa-M cells overexpressing Parkin is similar to that observed in hippocampal neurons expressing endogenous Parkin (30). E696K is a mutation in TBK1 that was previously shown to inhibit recruitment of TBK1 to mitochondria after depolarization (10). We treated E696K- or WT-TBK1–expressing cells with CCCP and compared the prevalence of TBK1 rings after 90 min (Fig. 1 *E* and *F* and *SI Appendix*, Fig. S2*B*). E696K-TBK1–expressing cells had significantly fewer rings/μm^2^ than WT-TBK1–expressing cells (Fig. 1*G*), establishing this approach as a quantitative measure of the functional effects of TBK1 on mitophagy. Of note, loss of mitochondrial mass was not observed within this time frame, as lysosomal degradation is not evident until ∼12 h after induction of mitochondrial damage (26, 27, 29).

### Dimerization Mutations Do Not Preclude TBK1 Recruitment.

**Fig. 2.**
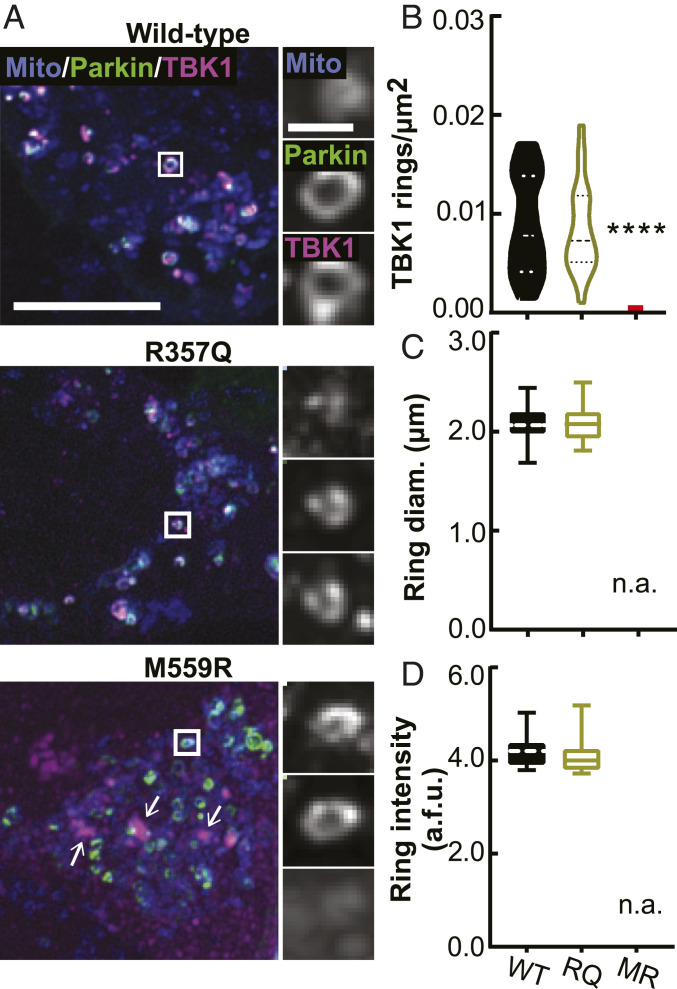
TBK1 mutants that are unable to dimerize are differentially recruited to damaged mitochondria. (*A*) Maximum intensity projection images of fixed HeLa cells depleted of endogenous TBK1 expressing a mitochondria-localized fluorophore (blue), Parkin (green), and WT- (*Top Row*), R357Q- (*Middle Row*), or M559R- (*Bottom Row*) TBK1 (magenta) fixed after 90 min CCCP. There are some aggregates of M559R-TBK1 (arrows) that are not coincident with mitochondria. (Scale bars: zoom out, 10 μm; zoom in, 2 μm.) Images shown are insets; for representative images of whole fields, reference *SI Appendix*, Fig. S2*C*. (*B*–*D*) Quantification of TBK1 rings/μm^2^ (*B*), ring diameter (*C*), and ring signal intensity (*D*). ****P < 0.0001 by ordinary one-way ANOVA with Dunnett’s multiple comparisons test. Dashed line, median; dotted lines, 25th and 75th quartiles. No M559R-TBK1 rings were evident, so all data points are 0 for rings/μm^2^ (red line), and no data can be displayed for size and intensity. *n* = 22 to 26 cells from three independent experiments. Data in *C* and *D* analyzed by Student’s unpaired *t* test. Not applicable, n.a. Arbitrary fluorescent units, a.f.u.

TBK1 dimerization is proposed to stabilize the trimodular structure of the molecule and permit efficient activation and kinase activity (13). We asked whether two ALS-associated mutations that prevent dimerization, R357Q and M559R (8), would affect TBK1 recruitment to damaged mitochondria. In basal conditions, SNAP-tagged R357Q- and M559R-TBK1 were cytosolic with intermittent puncta (*SI Appendix*, Fig. S1*F*), and their expression did not appreciably affect mitochondrial content. Following CCCP treatment, cells expressing WT-, R357Q-, or M559R-TBK1 exhibited robust Parkin recruitment to rounded mitochondria (Fig. 2*A* and *SI Appendix*, Fig. S2*C*). R357Q-TBK1 was recruited to the same extent as WT-TBK1 (Fig. 2 *A* and *B*). Strikingly, despite the higher cellular expression level (*SI Appendix*, Fig. S1*E*), no M559R-TBK1 recruitment to mitochondria was evident (Fig. 2 *A* and *B*). Instead, M559R-TBK1 remained largely cytosolic with some apparent aggregate formation, although these aggregates were not associated with mitochondria (Fig. 2*A*).

We measured the size and intensity of TBK1 rings to assess whether R357Q-TBK1 conferred a structural defect on the ubiquitin-based molecular platform that forms on damaged mitochondria. R357Q-TBK1 rings were the same diameter and average fluorescence intensity as WT-TBK1 rings (Fig. 2 *C* and *D*), indicating that the monomeric property of R357Q-TBK1 does not impair its ability to form the molecular ring structure. Together, these observations suggest that the lack of M559R-TBK1 recruitment is not due solely to an inability of the molecule to dimerize.

### Mutations Disrupting Both Dimerization and Activation Impair Recruitment of TBK1 to Damaged Mitochondria.

**Fig. 3.**
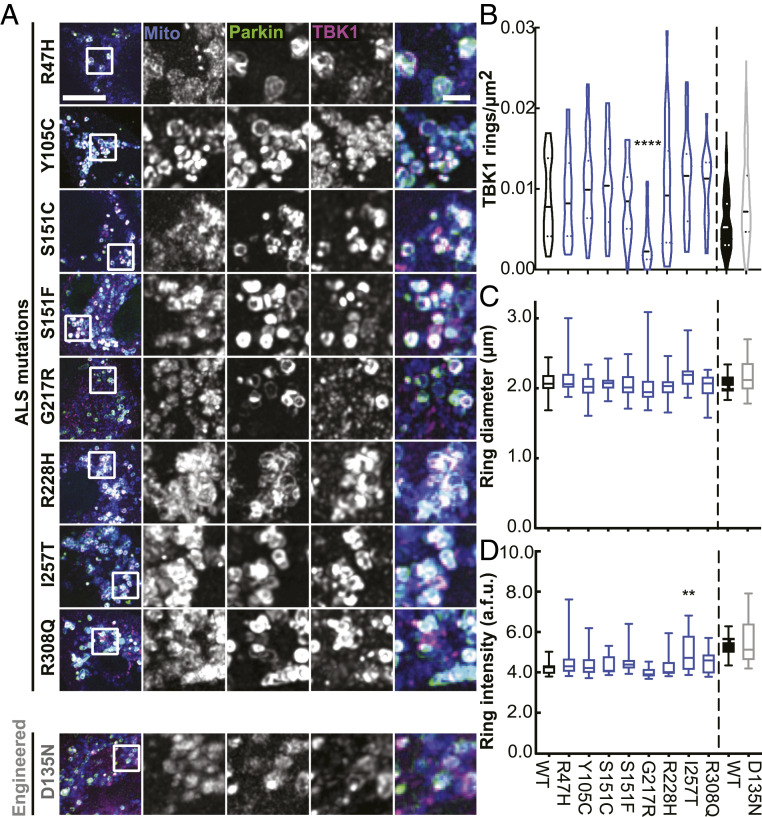
A kinase domain mutation that abolishes the autophosphorylation ability of TBK1 results in fewer TBK1 rings. (*A*) Maximum intensity projection images of fixed HeLa cells depleted of endogenous TBK1 expressing fluorescent mitochondrial marker (blue), Parkin (green), and TBK1 variants (magenta) and fixed after treatment with CCCP for 90 min. Images shown are insets; for representative images of whole fields, reference *SI Appendix*, Fig. S3*A*. (*B*–*D*) Quantification of TBK1 rings/μm^2^ (*B*), ring diameter (*C*), and ring signal intensity (*D*). WT-TBK1 ring data (indicated by black outlined plot and bars) are transferred from Fig. 2 for comparison. *n* = 22 to 32 cells from at least three independent experiments. For ring density, (*B*) horizontal dashed lines, median; horizontal dotted lines, 25th and 75th quartiles. For (*B*–*D*) vertical dashed lines distinguish separate data sets. **P < 0.01, ****P < 0.0001 by ordinary one-way ANOVA with Dunnett’s multiple comparisons test. Arbitrary fluorescent units, a.f.u.

The M559R mutation in TBK1 also disrupts kinase activation and enzymatic activity (Fig. 1*B*) (8), so we employed our mito-depolarization assay to test other TBK1 missense mutations that exhibit reduced autophosphorylation activity to varying degrees: R47H-TBK1, G217R-TBK1, and R228H-TBK1. G217R-TBK1 exhibits reduced dimer formation as well (8, 9). Of the mutants tested, only G217R-TBK1 exhibited deficient recruitment to damaged mitochondria compared to WT-TBK1. Expression of G217R-TBK1 resulted in significantly decreased TBK1 ring density, despite clear evidence of mitochondrial fragmentation and Parkin recruitment (Fig. 3 *A* and *B* and *SI Appendix*, Fig. S3*A*).

To further delineate the role of kinase activity, we expressed a TBK1 variant with an engineered mutation, D135N, which renders the TBK1 molecule kinase inactive and unable to autophosphorylate at S172 but fully able to dimerize (16). In line with previous data on an engineered phosphodeficient S172A-TBK1 mutant (10), D135N-TBK1 was recruited to damaged mitochondria to the same extent as WT-TBK1 (Fig. 3 *A* and *B*). Moreover, the ALS-associated mutations R47H-TBK1 and R228H-TBK1 have weaker autophosphorylation activity than WT-TBK1 based on biochemical studies (8), yet they also translocated to damaged mitochondria with the same incidence as WT-TBK1 (Fig. 3 *A* and *B*).

We saw no difference in either ring diameter or intensity across WT-, R47H-, G217R-, and R228H-TBK1 rings (Fig. 3 *C* and *D*) or when comparing WT- and kinase-inactive D135N-TBK1. Only I257T-TBK1, a kinase domain mutant exhibiting WT-TBK1 levels of autophosphorylation but weaker kinase activity toward OPTN, formed brighter rings than WT-TBK1. However, the average diameter of the I257T-TBK1 rings was not significantly different from WT-TBK1 rings (Fig. 3 *C* and *D*). Notably, the similarities between WT-TBK1 rings and the poorly recruited G217R-TBK1 rings demonstrate that expression of ALS-associated TBK1 mutants does not disrupt the integrity of TBK1 rings, even if fewer rings form. It is unlikely that mutant TBK1 recruitment is due to dimerization of mutant TBK1 with residual endogenous TBK1, since we measured knockdown levels >70% (*SI Appendix*, Fig. S1 *A* and *B*). To further substantiate this claim, we took advantage of the fact that M559R-TBK1–expressing cells exhibit no detectable recruitment of the tagged exogenous construct and probed CCCP-treated cells expressing M559R-TBK1 with an anti-TBK1 antibody to detect total TBK1. No TBK1 reactivity was detected at damaged mitochondria (*SI Appendix*, Fig. S3*B*).

To assess TBK1 recruitment with an alternative method of mitochondrial depolarization, we treated a subset of TBK1 variant-expressing cells with a combination of Antimycin A and Oligomycin A for 90 min (39). A majority of WT-, R357Q-, and D135N-TBK1–expressing cells exhibited TBK1 rings, while significantly less cells expressing G217R-TBK1 (43 ± 12%) or M559R-TBK1 (3.0 ± 3%) exhibited rings (*SI Appendix*, Fig. S4). Recapitulation of TBK1 recruitment patterns across different depolarizing insults fortifies our finding that R357Q- and D135N-TBK1 are recruited to damaged mitochondria to the same extent as WT-TBK1, while G217R- and M559R-TBK1 are deficient in this step of mitochondrial clearance.

### M559R- and G217R-TBK1 Display Disrupted Recruitment Kinetics Compared to WT- and R357Q-TBK1.

**Fig. 4.**
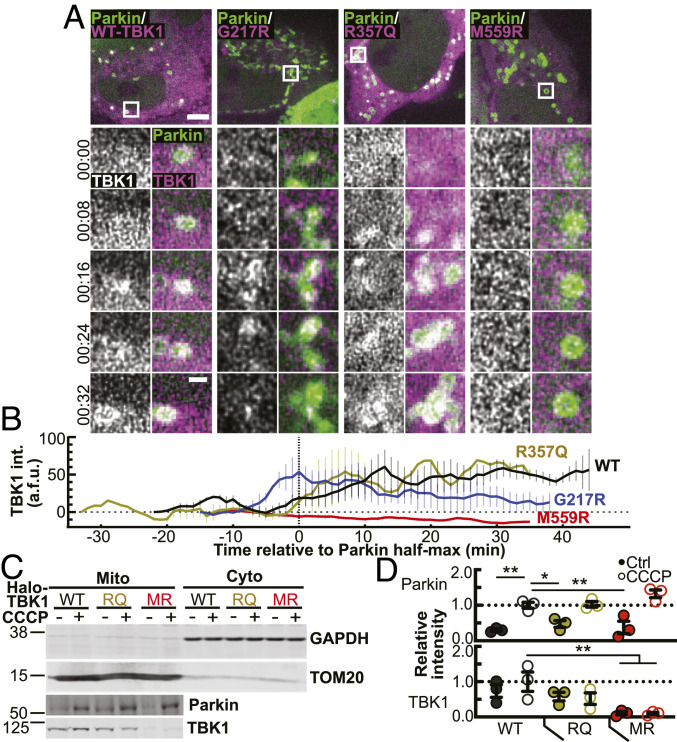
TBK1 variants exhibit differing kinetics and affinities with damaged mitochondria. (*A*) Representative confocal sections of live HeLa cells depleted of endogenous TBK1 expressing Parkin (green) and TBK1 variants (magenta), treated with CCCP for up to 90 min. White box (*Inset*) indicates a single representative event tracked over time to measure recruitment of Parkin and TBK1. Stills from timelapse are shown in the panels. Time is indicated as min:sec from initial Parkin recruitment. (Scale bars: zoom out, 10 μm; zoom in, 2 μm.) (*B*) Background-subtracted TBK1 signal intensity tracked over time with respect to Parkin half maximum (0, vertical dotted line). *n* = 3 to 6 representative events from at least three independent experiments. Error bars indicate SEM. (*C*) A representative Western blot of HEK TBK1^−/−^ cells expressing the respective TBK1 variants, treated with CCCP or vehicle, and enriched for mitochondria (*Left*) or cytosol (*Right*). Quantification was carried out on Mito fractions to compare association of the respective TBK1 variants and Parkin with mitochondria. Numbers to the left of blots indicate kDa. (*D*) Quantification of *C* with Parkin (*Top*) and TBK1 (*Bottom*) bands normalized to TOM20 and compared to average level of WT-TBK1–expressing cells treated with CCCP (dotted line). *P ≤ 0.05, **P < 0.01 by ordinary one-way ANOVA with Dunnett’s multiple comparisons test. Error bars indicate SEM. *n* = 3 independent experiments.

Biochemical analyses indicate that the missense mutations G217R, R357Q, and M559R impair the function of TBK1 (3, 8, 9). Expression of these variants may disrupt recruitment kinetics during individual mitophagic events, as compared to WT-TBK1. We performed live-cell microscopy using Halo-tagged TBK1 constructs and tracked single mitophagy events from initial Parkin recruitment to peak TBK1 recruitment in cells expressing similar levels of exogenous TBK1. R357Q-TBK1 exhibited the same kinetics as WT-TBK1 (Fig. 4*A*), reaching maximum intensity as a fully formed ring ∼10 to 15 min after Parkin reached its half maximum. Together, the comparative kinetics (Fig. 4*B*) and ring prevalence between WT- and R357Q-TBK1 (Fig. 2) suggest that TBK1 dimerization is not required for recruitment and assembly of TBK1 at the depolarized mitochondria.

G217R-TBK1 translocated to and coalesced at damaged mitochondria in bright but unstable structures at around the same time that Parkin reached its half maximum (Fig. 4 *A* and *B*). These unstable configurations reached the same raw maximum intensity as WT- and R357Q-TBK1 and occasionally appeared as full rings. Some G217R-TBK1 rings remained intact over the course of our observation (up to 60 min), corroborating our results in fixed cells (Fig. 3*A*), but the majority disappeared within 20 min of reaching peak intensity (Fig. 4*B*). In contrast, over the course of 90 min of mitochondrial damage M559R-TBK1 was never recruited to damaged mitochondria (Fig. 4 *A* and *B*).

The kinetic data (Fig. 4 *A* and *B*) and the results from fixed cells (Figs. 2 and 3) indicate that there is a specific window of time during which TBK1 must be recruited in order to properly form and maintain a stable ring. If such interactions are insufficient, TBK1 molecules disperse from the damaged organelle as we see with G217R-TBK1. The inability of G217R-TBK1 to carry out autophosphorylation combined with its reduced dimerization may impede the stable interaction with ubiquitinated mitochondria. Since R357Q-TBK1 can be phosphorylated and activated even as a monomer, it was able to maintain a stable ring structure. M559R-TBK1 is completely monomeric, and it is even less likely to stably interact with the mitochondria, thus there are no detectable rings following mitochondrial damage.

To further probe the integrity of the association of TBK1 with damaged mitochondria, we employed a mitochondrial fractionation assay. We expressed R357Q-, M559R-, or WT-TBK1 in human embryonic kidney (HEK) cells in which both TBK1 alleles had been deleted by CRISPR-Cas9 (HEK TBK1^−/−^) (8), then enriched mitochondria from the cells after CCCP or vehicle treatment (Fig. 4*C*). CCCP treatment resulted in twice as much Parkin enriched in the mitochondrial fraction of each sample as compared to vehicle treated cells, demonstrating the assay’s sensitivity to Parkin-dependent mitophagy. This Parkin enrichment corroborated our immunofluorescent data in HeLa-M cells as well as previous experiments in HEK cells (38). Expression of R357Q- or M559R-TBK1 did not affect Parkin enrichment after mitochondrial depolarization.

TBK1 is recruited to OPTN puncta after 90 min CCCP treatment in HEK cells, however the effect is less robust than in HeLa cells (*SI Appendix*, Fig. S4*C*). In mitochondrial fractions, there was a low affinity association of WT- and R357Q-TBK1 with mitochondria under basal conditions (Fig. 4 *C* and *D*). In contrast, M559R-TBK1 was barely present in the mitochondrial fraction (Fig. 4 *C* and *D*). With CCCP treatment, we did not detect a significant increase in TBK1 in the mitochondrial fraction with expression of any of the variants, suggesting that TBK1 may be transiently associated with mitochondria even in the absence of induced stress. The absence of M559R-TBK1 under basal or mitochondrial damage conditions substantiates the severe functional defect of M559R-TBK1 seen in HeLa assays.

### OPTN Phosphorylation Is Enhanced by TBK1 Recruitment but Not Fully Dependent on TBK1 Activity.

Activated TBK1 leads to the phosphorylation of OPTN on at least two residues, S177 and S513; these phosphorylation events enhance the binding of OPTN to ubiquitin chains deposited on damaged mitochondria in the early stage of mitophagy (18). However, TBK1 activity is not required for OPTN recruitment to damaged mitochondria, as OPTN was recruited in cells in which TBK1 was either depleted or its kinase activity was inhibited (10) (*SI Appendix*, Fig. S5*A*). Furthermore, we found that none of the TBK1 mutants affected OPTN recruitment to damaged mitochondria (*SI Appendix*, Fig. S5*B*). In the clearest case, expression of M559R-TBK1 results in a complete absence of TBK1 rings (Fig. 2). However, OPTN ring prevalence was not impacted, and TBK1 mutant expression did not cause variations in the size or intensity of OPTN rings, with the exception of a slightly larger diameter of OPTN rings induced by R47H-TBK1 expression (*SI Appendix*, Fig. S5 *B* and *C*).

**Fig. 5.**
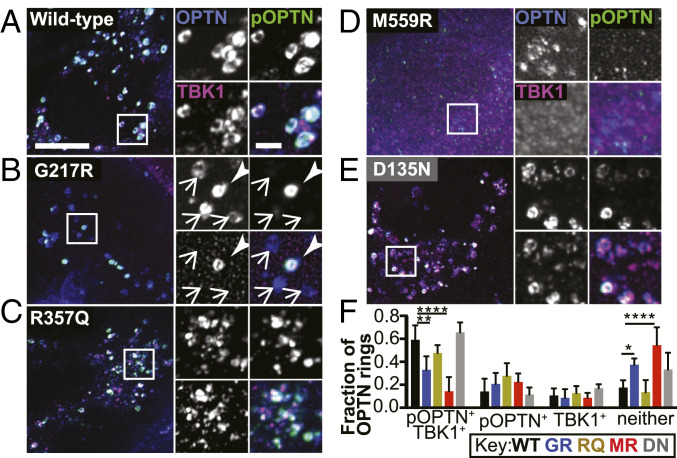
ALS-linked TBK1 mutants suppress OPTN phosphorylation. (*A*–*E*) Maximum intensity projection images of HeLa cells depleted of endogenous TBK1 and expressing Parkin (not tagged), OPTN (blue), and WT- (*A*), G217R- (*B*), R357Q- (*C*), M559R- (*D*), or D135N- (*E*) TBK1 variants (magenta), fixed after treatment with CCCP for 90 min. Phospho-S177 OPTN is tagged with an antibody (green). In *B*, one ring is positive for phospho-OPTN and TBK1 (arrowhead), and the others are negative for both (arrows). (Scale bars: zoom out, 10 μm; zoom in, 2 μm.) Images shown are insets; for representative images of whole fields, reference *SI Appendix*, Fig. S6*A*. (*F*) For each cell, the percentage of OPTN rings in each category was calculated. Error bars indicate SD. *n* = 8 to 15 cells from at least three independent experiments. *P ≤ 0.05, **P < 0.01, ****P < 0.0001 by ordinary one-way ANOVA with Dunnett’s multiple comparisons test.

The LC3-interacting region of OPTN is involved in facilitating formation of the LC3-positive autophagosome (35), so both recruitment and phosphorylation of OPTN at S177 may be necessary to complete clearance of damaged mitochondria. We asked whether expression of mutant variants of TBK1 inhibits the phosphorylation of OPTN. To this end, we performed immunofluorescence using an antibody to phospho-S177 OPTN to assess the extent of OPTN modification. WT-TBK1–expressing cells exhibited robust OPTN rings after 90 min of CCCP treatment (Fig. 5*A* and *SI Appendix*, Fig. S6*A*). Furthermore, WT-TBK1 colocalized with OPTN rings, and there was a strong phospho-OPTN signal coincident with TBK1-positive OPTN rings (Fig. 5*A*). Over 75% of OPTN rings in each cell were either TBK1 positive, phospho-OPTN positive, or positive for both; the majority (58 ± 5.8%) of all OPTN rings were coincident with both TBK1 and phospho-OPTN (Fig. 5*F*). R357Q- and D135N-TBK1 expression exhibited similar results (Fig. 5 *C, E,* and *F*).

G217R- and M559R-TBK1–expressing cells exhibited OPTN rings with lower intensities of TBK1 and phospho-OPTN (*SI Appendix*, Fig. S6 *B* and *C*). Consistent with our observations that G217R-TBK1 rings were not as prevalent as WT-TBK1 rings (Fig. 3), G217R-TBK1–expressing cells exhibited fewer OPTN rings that were positive for TBK1. However, ∼50% of OPTN rings were positive for phospho-OPTN (Fig. 5*B*). With M559R-TBK1 expression, a minority of OPTN rings were positive for TBK1, phospho-OPTN, or both (Fig. 5 *D* and *F*). The evidence that any phospho-OPTN is present in D135N-, G217R-, and M559R-TBK1–expressing cells is surprising given the in vitro finding that TBK1 with these mutations cannot carry out phosphorylation (8), even while it can be recruited to ubiquitinated mitochondria (Fig. 3*B*). While we cannot rule out contributions from residual levels of endogenous TBK1, it may also be the case that another kinase can phosphorylate OPTN in the absence of TBK1 activity.

**Fig. 6.**
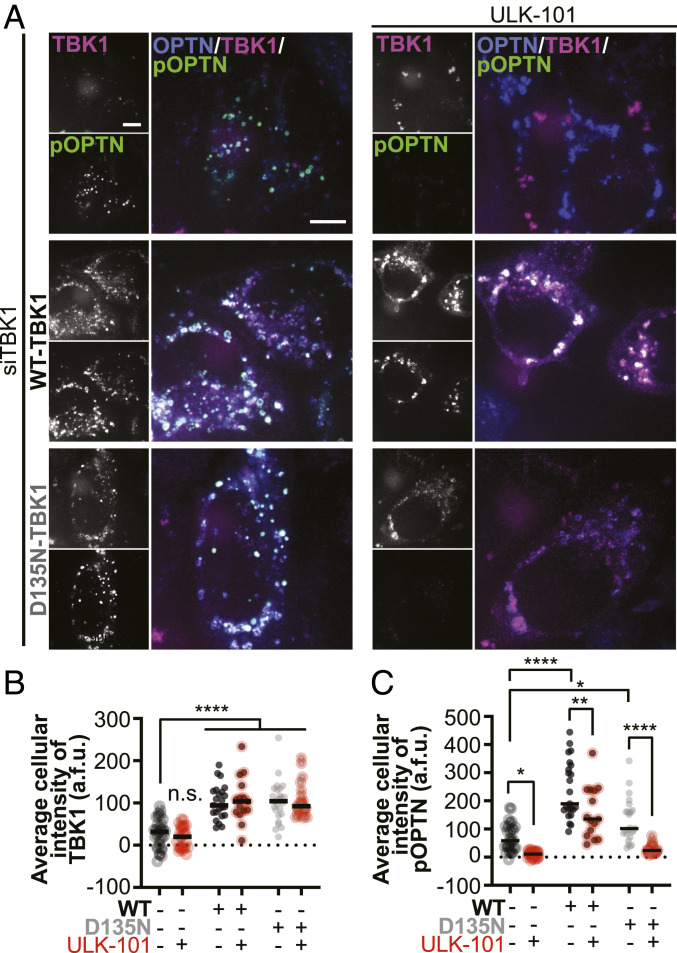
ULK1 contributes to OPTN phosphorylation independent of TBK1 kinase activity. (*A*) Maximum intensity projection images of HeLa cells depleted of endogenous TBK1 and expressing Parkin (not tagged) and OPTN (blue). Phospho-S177-OPTN was labeled with an antibody (green). In the *Top Row*, cells were not rescued with exogenous TBK1; magenta channel shows fluorescent ligand alone. In the *Middle* and *Bottom Rows* cells were rescued with WT- and D135N-TBK1, respectively (magenta). Half of each set was treated with the ULK1 complex inhibitor ULK-101 (*Right Column*) and all were fixed after treatment with CCCP for 90 min. (Scale bars, 8 μm.) (*B* and *C*) Whole-cell average intensities of TBK1 (*B*) or pOPTN (*C*) signal after background subtraction was measured for each condition. Bars indicate medians. *n* = 8 to 15 cells from at least three independent experiments. n.s., not significant (n.s. where not specified), *P ≤ 0.05, **P < 0.01, ****P < 0.0001 by two-way ANOVA with multiple comparisons.

There is a high structural similarity between the N-terminal domain of FAK family kinase-interacting protein of 200 kDa (FIP200) and the scaffolding dimerization domain of TBK1. In forming the ULK1 complex, the component kinase ULK1 associates with FIP200 in an analogous position to the kinase domain of TBK1 (33). Thus, we wondered if the ULK1 complex could contribute to phosphorylation of OPTN during mitophagy. We inhibited the ULK1 complex with a specific inhibitor, ULK-101 (40) in cells depleted of TBK1 and expressing WT-TBK1, the engineered kinase-inactive mutant D135N-TBK1, or with no rescue (Fig. 6*A*).

Corroborating our earlier results (Fig. 3), WT-TBK1 and D135N-TBK1 were recruited to the same extent after mitochondrial damage, as measured by whole-cell average intensities of TBK1 (Fig. 6*B*). Treatment with ULK-101 did not affect TBK1 recruitment. However, the persistent levels of phospho-S177 OPTN observed even in cells depleted of TBK1 (Fig. 6*C*) was abrogated upon treatment with ULK-101. As expected, cells expressing WT-TBK1 exhibited higher levels of phospho-S177 OPTN after CCCP treatment; however, ULK-101 treatment diminished the average intensity of phospho-OPTN. Phospho-OPTN intensity was higher in cells expressing D135N-TBK1 compared to cells depleted of TBK1, indicating that recruitment of inactive TBK1 is sufficient to induce OPTN phosphorylation. Strikingly, inhibition of ULK1 in D135N-TBK1–expressing cells reduced phospho-OPTN to the same level seen in TBK1-depleted cells treated with the ULK1 inhibitor. These results suggest that ULK1 complex activity contributes to OPTN phosphorylation. Furthermore, since expression of kinase-inactive D135N-TBK1 was sufficient to increase phospho-OPTN levels, we hypothesize that TBK1 recruitment facilitates ULK1 activity, leading to phosphorylation of OPTN, albeit to a lower extent. Thus, we propose that ALS-linked mutants G217R- and M559R-TBK1 diminish phospho-OPTN levels because they are not recruited to damaged mitochondria.

### TBK1 and Phospho-OPTN Are Both Required for Efficient LC3 Recruitment.

**Fig. 7.**
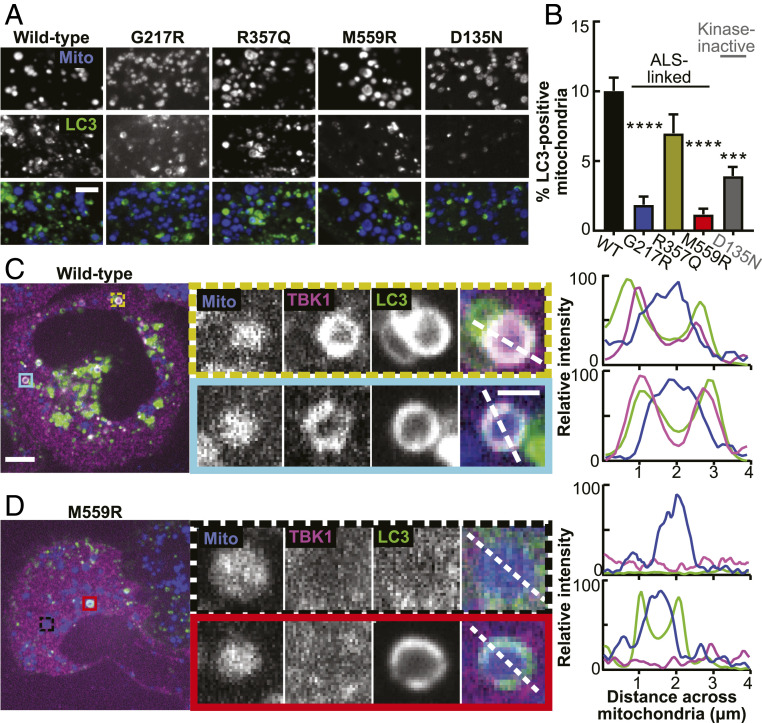
TBK1 recruitment and phosphorylation of OPTN are both necessary for efficient mitochondrial engulfment by the LC3-positive autophagosome. (*A*) Representative confocal images of fixed HeLa cells depleted of endogenous TBK1 and expressing Parkin (not tagged), a fluorescent mitochondrial marker (blue), LC3 (green), and TBK1 (respectively indicated above each column), fixed after treatment with CCCP for 90 min. (Scale bar, 3 μm.) (*B*) Percent of LC3-positive mitochondria in cells expressing the respective TBK1 mutants. *n* = 5 to 15 cells from at least three independent experiments. ***P < 0.001, ****P < 0.0001 by ordinary one-way ANOVA with Dunnett’s multiple comparisons test. Error bars indicate SEM. (*C* and *D*) Representative confocal images of fixed HeLa cells depleted of endogenous TBK1 and expressing Parkin (not tagged), a fluorescent mitochondrial marker (blue), WT- (*C*) or M559R- (*D*) TBK1 (magenta), and LC3 (green) fixed after treatment with CCCP for 90 min. *Insets* (colored boxes and zoom panels) display examples of mitophagy events. The adjacent traces (*Right*) display quantification of relative signal intensity of each channel over a line scan (white dashed lines) across the diameter of the rounded mitochondria. (Scale bars: zoom out, 10 μm; zoom in, 2 μm.) Images shown in *A*, *C*, and *D* are insets; for representative images of whole fields, reference *SI Appendix*, Fig. S7.

The sequence of molecular events investigated thus far serves to induce formation of a double membraned autophagosome classically identified by membrane-associated LC3 (41). After sufficient expansion, the membrane engulfs the damaged organelle (*SI Appendix*, Fig. S1*D*) before fusing with acidic lysosomes to complete the degradation process. In order to test whether TBK1 mutant variants would alter autophagosome engulfment, we quantified the percentage of LC3-positive mitochondria after CCCP treatment. With WT-TBK1 expression, clear recruitment of LC3 was observed to 10 ± 0.9% of damaged mitochondria in a single confocal section at the 90 min time point and could be visualized as rings coincident with TBK1 (Fig. 7 *A*–*C* and *SI Appendix*, Fig. S7). Expression of the monomeric R357Q-TBK1 did not result in a statistically significant change in percent of mitochondria engulfed in LC3 rings (Fig. 7*B*).

In contrast, expression of G217R-TBK1 and M559R-TBK1 mutants significantly inhibited the formation of LC3 rings on damaged mitochondria, with 1.9% ± 0.6 and 1.2% ± 0.4 LC3-positive mitochondria, respectively (Fig. 7 *A* and *B*). Interestingly, while no M559R-TBK1 rings were detectable, some mitochondria in M559R-TBK1–expressing cells were LC3 positive (Fig. 7 *D*, *Bottom Inset*). The formation of LC3-positive mitochondria even in the absence of TBK1 can potentially be explained by a compensatory mechanism in which a different mitophagy receptor, NDP52, is recruited to ubiquitinated mitochondria independently of OPTN and TBK1 and can recruit LC3 by an alternative mechanism (10). However, this alternative mechanism is less efficient, as there were many fewer LC3-positive mitochondria.

Expression of the kinase-inactive D135N-TBK1 construct also impaired LC3 recruitment to damaged mitochondria. There were significantly fewer LC3-positive mitochondria with D135N-TBK1 rescue (3.9% ± 0.65) compared to WT-TBK1 (Fig. 7*B*). The residual autophagosome formation observed in cells expressing the kinase-inactive variant is consistent with our data that recruitment of inactive TBK1 facilitates partial, ULK1-dependent OPTN phosphorylation. We conclude that TBK1 recruitment to damaged mitochondria is required and that one of the key targets of TBK1, OPTN, must be phosphorylated in order to activate autophagosomal formation in an efficient manner.

### Expression of ALS-Associated TBK1 Mutants Alters Mitochondrial Network Health and Sensitivity to Oxidative Stress.

Given that TBK1 missense mutations differentially affected mitophagy when expressed in HeLa-M cells, we asked how expression of these mutants would affect mitochondrial homeostasis in primary neurons. We depleted endogenous TBK1 from primary rat hippocampal neurons and expressed constructs encoding WT-, R357Q-, or M559R-TBK1 (*SI Appendix*, Fig. S8 *A*–*C*). While the R357Q- and M559R-TBK1 constructs were expressed in a lower percentage of neurons than WT-TBK1, expression within individual neurons was similar for all constructs examined as quantified by cellular fluorescence intensity measurements (*SI Appendix*, Fig. S8*D*).

**Fig. 8.**
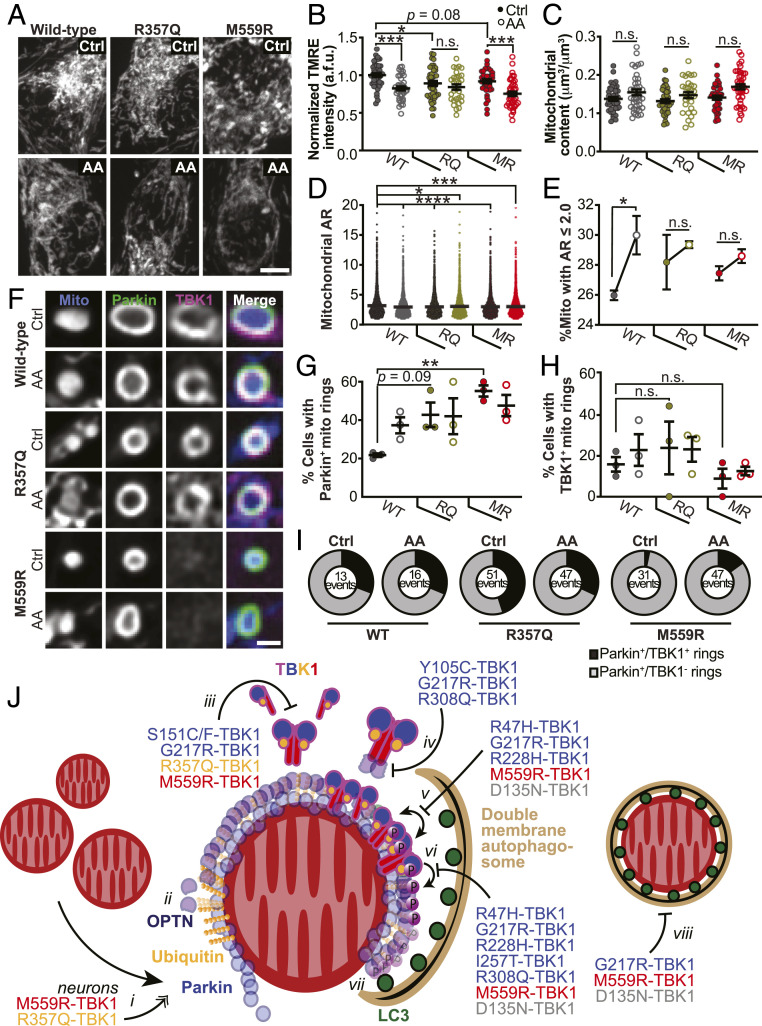
Expression of ALS-associated TBK1 mutants alters mitochondrial network health and sensitivity to oxidative stress, and a model for the deleterious effects of TBK1 mutations in mitophagy. (*A* and *B*) Representative images (*A*) and quantification (*B*) of TMRE fluorescence intensity. Control basal conditions, Ctrl. Mean ± SEM; *n* = 30 to 42 neurons from three to four biological replicates; 7 days in vitro (DIV). Not significant, n.s.; *P ≤ 0.05, ***P < 0.001 by one-way ANOVA with Sidak’s multiple comparisons test. (Scale bar, 5 μm.) (*C*) Quantification of mitochondrial content by volume. Mean ± SEM; *n* = 30 to 42 neurons from three to four biological replicates; 7 DIV. Not significant, n.s., by Kruskal–Wallis ANOVA with Dunn’s multiple comparisons test. (*D*) Quantification of mitochondrial AR for all mitochondria observed. Mean ± SEM; *n* = 30 to 42 neurons from three to four biological replicates; 7 DIV. *P ≤ 0.05, ***P < 0.001, ****P < 0.0001 by one-way ANOVA with Dunnett’s multiple comparisons test. (*E*) Percent of mitochondria with a mitochondrial AR ≤2. Mean ± SEM; *n* = 30 to 42 neurons from three to four biological replicates; 7 DIV. Not significant, n.s.; *P ≤ 0.05 by one-way ANOVA with Sidak’s multiple comparisons test. (*F*) Representative images of Parkin-positive mitochondria with examples that are TBK1 positive (WT and R357Q) and TBK1 negative (M559R). (Scale bar, 1 μm.) (*G* and *H*) Quantification of the percent of neurons with Parkin-positive (*G*) or TBK1-positive (*H*) mitochondria rings. Mean ± SEM; *n* = 25 to 32 neurons from three biological replicates; 7 DIV. Not significant, n.s.; **P < 0.01 by one-way ANOVA with Sidak’s multiple comparisons test. (*I*) Quantification of the number of Parkin-positive mitochondria (total) that are TBK1 positive (black sector) or TBK1 negative (gray sector). *n* = 25 to 32 neurons from three biological replicates; total number of events are shown; 7 DIV. (*J*) Model for TBK1 involvement in mitophagy and effects of mutants. *i*) Upon mitochondrial depolarization, Parkin (blue circles) is recruited to the OMM and ubiquitinates (gold spheres) outer membrane proteins. In neurons, expression of R357Q- or M559R-TBK1 induces more rounded, Parkin-positive mitochondria. *ii*) Ubiquitin chains recruit OPTN (purple circles), which interact with ubiquitin via their UBAN domains. TBK1 is not required for this interaction. *iii*) TBK1 (multicolored cartoon) monomers constitutively dimerize along their scaffolding dimerization domains. Five mutations disrupt this dimerization, including R357Q-TBK1 and M559R-TBK1, which have completely disrupted dimerization. *iv*) TBK1 associates with OPTN at its CTD, thus TBK1 may be corecruited with OPTN to the mitophagy site. Three ALS-linked mutations in TBK1 exhibit disrupted OPTN association, yet Y105C-TBK1 and R308Q-TBK1 can still be recruited to the damaged mitochondria; thus TBK1 can also be independently recruited. *v*) Formation of TBK1 multimers at the mitochondria surface promotes TBK1 transautophosphorylation, by which TBK1 is activated upon phosphorylation at S172 (purple circles with “P”). Four ALS-linked TBK1 mutants and the engineered D135N-TBK1 have diminished or abolished activation. *vi*) Activated TBK1 phosphorylates the mitophagy receptor OPTN at S177. Activated TBK1 may also promote autophagosomal membrane expansion (tan crescent). *vii*) Phosphorylated OPTN is necessary to recruit the LC3-coated (dark green circles) autophagosome. *viii*) The double membrane autophagosome completely engulfs a damaged mitochondria.

First, we examined mitochondrial network health under basal conditions, focusing on somal mitochondria as our previous work has shown most mitophagic events occur within this compartment (30). We assessed network health using the polarization-dependent mitochondrial dye, tetramethylrhodamine ethyl ester (TMRE) (Fig. 8 *A*, *Top*). TMRE intensities for neurons expressing R357Q-TBK1 were significantly reduced compared to WT-TBK1 under basal conditions, indicating that mutant expression is sufficient to negatively impact network health (Fig. 8 *A* and *B*). Of note, there was no correlation between levels of TBK1 expression and TMRE intensity at the cellular level (*SI Appendix*, Fig. S8*D*) and no loss of somal mitochondrial mass in neurons expressing any of the variants under these conditions (Fig. 8*C*).

We then induced mitophagy in hippocampal neurons by applying 3 nM Antimycin A (AA) over 2 h (Fig. 8 *A*, *Bottom*), as performed previously (30). Following treatment with AA, neurons expressing either WT- or M559R-TBK1 exhibited significantly lower intensities of TMRE (Fig. 8 *A* and *B*) than were observed under basal conditions. The TMRE intensity of the somal mitochondrial network in R357Q-TBK1–expressing neurons did not significantly decrease with AA treatment (Fig. 8*B*). As an additional measure of mitochondrial damage, we analyzed mitochondrial morphology by measuring the aspect ratios (ARs) of mitochondria in neurons expressing WT-, R357Q-, or M559R-TBK1 under control conditions or when treated with AA. All conditions exhibited significantly decreased ARs as compared to mitochondria in neurons expressing WT-TBK1 under basal conditions (Fig. 8*D*), evidence that either mitochondrial depolarization with AA or expression of mutant TBK1 is sufficient to alter mitochondrial network properties. As mitochondrial rounding is a measure of stress, we focused specifically on the percentage of mitochondria with an AR of ≤2. Mutant-expressing cells tended to exhibit more rounded mitochondria than WT-expressing cells in basal conditions, corroborating our observation that mutant-expressing neurons exhibit mitochondrial stress (Fig. 8 *B* and *E*). Given that mitochondria were already more rounded with expression of mutant TBK1 under control conditions, only neurons expressing WT-TBK1 exhibited a significant increase in rounding after AA-induced mitochondrial depolarization (Fig. 8*E*).

Depolarized mitochondria effectively recruited both Parkin and TBK1 in neurons expressing WT-TBK1 (Fig. 8*F*) as expected (30). In striking contrast, expression of either R357Q- or M559R-TBK1 was sufficient to cause increased Parkin recruitment to mitochondria even under basal conditions, while mitochondrial depolarization by AA did not further increase the number of Parkin rings associated with somal mitochondria (Fig. 8 *F* and *G*). Since transient Parkin expression may up-regulate mitophagy and affect TBK1 recruitment in neurons, we identified cells with fewer than 10 Parkin-positive mitochondria per soma to measure whether there was TBK1 recruitment to these events. In total, 20 to 30% of neurons expressing WT- and R357Q-TBK1 exhibited mitochondria that recruited TBK1 (Fig. 8*H*). Despite 50 to 60% of M559R-TBK1–expressing neurons exhibiting Parkin-positive rings (Fig. 8*G*), only 10 to 15% of cells had TBK1-positive rings (Fig. 8*H*). We looked more closely at individual events, in which depolarized, rounded mitochondria recruited Parkin (Fig. 8 *F* and *I*). R357Q-TBK1, like WT-TBK1, was recruited to one third of all Parkin-positive mitochondria, while M559R-TBK1 was recruited to less than a tenth of Parkin-positive mitochondria (Fig. 8*I*). Though R357Q-TBK1 was recruited to the same proportion of Parkin-positive events as WT-TBK1 (Fig. 8*I*), we observed a greater number of total events in R357Q-TBK1—expressing neurons (Fig. 8*G*). This increase may indicate that the monomeric property of R357Q-TBK1 induces less efficient interactions with depolarized mitochondria in neurons, leading to a glut of stalled mitophagy events. In contrast, M559R-TBK1 expression results in loss of TBK1 recruitment, (Fig. 8 *F*, *H*, and *I*) which may result in a more severe mitophagy defect.

## Discussion

Here, we present a functional analysis of recently identified and characterized ALS- and ALS-FTD–linked TBK1 mutations (8). Intriguingly, these mutations are located throughout the structure of the TBK1 molecule and result in diverse biochemical consequences, with differential effects on dimerization, autophosphorylation, OPTN association, and kinase activity. While TBK1 mutations may contribute to neurodegenerative pathogenesis through a number of different pathways, our study compares the effects of many of these mutations on the clearance of damaged mitochondria via mitophagy (Fig. 8*J*), a process thought to be crucial to maintaining neuron health. We build upon previous work to propose a model of TBK1 activity in mitophagy that reinforces the hypothesis that disordered mitochondrial clearance plays a role in the development of ALS.

Previous work has suggested that TBK1 dimerization promotes kinase activation (13–15). However, most of the TBK1 mutants known to disrupt dimerization were recruited to damaged mitochondria and formed rings with the same robustness as WT-TBK1. Notably, the ubiquitin-like domain mutant R357Q-TBK1 was shown to be fully monomeric (8), yet R357Q-TBK1 rings formed with the same prevalence as WT-TBK1 rings in HeLa-M cells, and R357Q-TBK1 was recruited to the same proportion of Parkin-positive mitochondria in hippocampal neurons. In hippocampal neurons, however, expression of R357Q-TBK1 was sufficient to induce mitochondrial stress and fragmentation in both basal and oxidative stress conditions. Thus, even as the effects of inhibited dimerization on mitophagy are subtle in HeLa cells, they become magnified in a more specialized cell type.

OPTN depletion prevents TBK1 recruitment to damaged mitochondria and inhibits efficient autophagosome formation (10). Interestingly, the TBK1 mutations Y105C and R308Q reduce association of TBK1 with OPTN (8), yet both mutants were recruited to damaged mitochondria. Thus, TBK1 association with its adaptors may be more complex than previously thought. TBK1 interacts in a mutually exclusive manner with OPTN or another adaptor at its CTD; however, the C-terminal TBK1 mutation E696K affects only OPTN association and not NAP1 association (7, 16). Future experiments should investigate whether ALS-TBK1 mutations predispose TBK1 to associate with NAP1, Sintbad, or TANK instead of OPTN, or vice versa, and how this balance could impact the functional roles of TBK1.

TBK1 recruitment is thought to be necessary for OPTN phosphorylation, which enhances the affinity of OPTN for ubiquitin chains (7, 18) and is required for its interaction with LC3 (10, 18, 27). We found that partial phosphorylation of OPTN was observed even with expression of mutants that abolished TBK1 recruitment. We used the ULK1 inhibitor ULK-101 to demonstrate that this limited OPTN phosphorylation is dependent on ULK1 complex activity, suggesting that the ULK1 kinase may directly phosphorylate OPTN. This finding suggests that the kinase-dependent regulation of mitophagy is not simply a linear pathway, but instead the kinase activities of TBK1 and ULK1 are interdependent to some degree, a possibility that will require further work to explore.

Generation, recruitment, and engulfment by the LC3-marked phagophore is the final step before the new mitophagosome compartment fuses with acidic lysosomes. Expression of the R357Q-TBK1 mutation, which is recruited to damaged mitochondria and has a functional kinase despite its monomeric form, promotes LC3 recruitment to damaged mitochondria at WT levels in HeLa-M cells. Expression of the ALS-associated G217R and M559R mutations leads to significantly fewer LC3-positive damaged mitochondria after global oxidative damage. However, our data show that phospho-OPTN is associated with damaged mitochondria in G217R- and M559R-TBK1–expressing cells with the same prevalence as is found in WT-TBK1–expressing cells. Thus, our findings highlight the requirement for both TBK1 kinase activity and TBK1 recruitment in order to promote autophagosomal engulfment of damaged mitochondria.

TBK1 mutants that did not measurably affect mitophagy in these experiments may induce more subtle defects that only emerge over a longer period of time or in specialized cells, as we saw with the differing effects of R357Q-TBK1 expression between HeLa-M cells and hippocampal neurons. This disparity could be due to uniquely sensitive roles of the protein in different cell types; alternatively, some of the missense mutations in TBK1 may induce misfolding or protein instability and thus decreased expression levels. Previous work has shown that some heterozygous mutations in TBK1 produce premature stop codons, frameshifts, or in-frame deletions and cause ALS by haploinsufficiency (3). We performed imaging analyses on cells with similar expression levels of tagged TBK1 constructs (*SI Appendix*, Figs. S2 *A* and *B* and S8*D*); however, we did note that cell lysate analyses revealed some of the mutants examined were poorly expressed compared to WT-TBK1 (*SI Appendix*, Figs. S2*C* and S8*C*). While in HeLa-M cells, some inefficiencies might be compensated by a high concentration of mutant TBK1, in neurons, a lower availability of the monomeric R357Q-TBK1 could be unable to form the proper scaffold needed for ULK1 complex regulation. This would lead to deficient phosphorylation of OPTN and inadequate recruitment of the autophagosome, despite the equivalent kinase activity of R357Q to WT TBK1. It will be important to examine endogenous expression levels of TBK1 in patient-derived material to more accurately distinguish between loss of function and haploinsufficiency.

Finally, though our study focuses on TBK1 recruitment kinetics and phosphorylation of OPTN, recent work points to other roles for TBK1 within mitophagy. TBK1 also phosphorylates RAB7A to recruit ATG9-positive vesicles as a source of autophagosomal membrane (42), facilitates the interaction of NDP52 with the FIP200/ULK1 complex to promote ULK1 activation (43), and phosphorylates LC3C and GABARAP-L2 to ensure a steady availability of the autophagosome membrane (44), each of which may also be affected by TBK1 mutations. It is also possible that mutations in TBK1 disrupt other critical pathways, such as inflammation. In the NF-κB response pathway, TBK1 is required to interact with TNF receptor–associated factor 2 (TRAF2) and TANK (45); this network may be hindered by missense mutations in TBK1. Inflammatory and viral response mechanisms may intersect or converge with mitophagy or be wholely separate from the pathway of mitochondrial clearance, leading to varying presentations of the same disease (46, 47). Interestingly, two independent patients with the same TBK1 mutation presented with similar phenotypes of ALS (3), pointing toward the merit of further systematic correlation of genetic mutations with disease presentations.

Overall, our results indicate that some TBK1 mutations disrupt mitophagic flux, inhibiting or delaying clearance of the damaged organelles. We also noted that TBK1 mutant expression in primary neurons was sufficient to induce stress within the mitochondrial network. The accumulation of dysfunctional mitochondria may deplete cellular energy pools and/or produce cytotoxic reactive oxygen species (ROS), triggering neurodegeneration. It is also possible that a disruption in flux could lead to sequestration of OPTN, TBK1, or other mitophagy components on damaged mitochondria, preventing these proteins from carrying out other cellular roles (48, 49). Deficient mitophagy may also stimulate innate immune pathways (50, 51) and promote buildup of toxic aggregates (52), provoking neuroinflammation, another hallmark of ALS (53). Critical questions persist regarding how dysfunctional mitochondria, neuroinflammation, and toxic aggregates relate to one another in the pathogenesis of ALS. Moreover, the relative roles of neural and glial dysfunction, age of onset, and exacerbating factors such as a “second hit” (54, 55) must be explored. Further dissection of those phenomena will orient our approach to therapeutic development in the future.

## Materials and Methods

Mutated TBK1 constructs were generated as described in Ye et al. (8) SNAP-tagged and Halo-tagged versions of TBK1 constructs were generated by inserting SNAP (pSNAPf [New England Biolabs]) or Halo (pHaloTag vector, Promega) at the N terminus of the TBK1 coding region. HeLa-M or HEK293T cells were transfected with exogenous constructs 24 h before sample collection. Hippocampal neurons were transfected 36 to 48 h before imaging and collection. Mitochondrial enrichment was performed with ThermoScientific isolation kit for cultured cells (89874). HeLa-M cells and neurons were labeled with fluorescent ligands prior to treatment. Where applicable, fixation was done with 4% paraformaldehyde after CCCP treatment. Confocal microscopy was performed on an UltraView Vox spinning disk confocal system and images were deconvolved with Huygens Professional Software, then analyzed with ImageJ/FIJI, Ilastik, and CellProfiler software (56–58). Notably, intensity measurements were collected from original data not deconvolved images. For details regarding all materials and methods, reference *SI Appendix*.

## Supplementary Material

Supplementary File

## Data Availability

Original data have been deposited in Zenodo (https://doi.org/10.5281/zenodo.4670341) (59).
