# Supplementary material for: ALS- and FTD-associated missense mutations in TBK1 differentially disrupt mitophagy
Source: Proc Natl Acad Sci U S A. 2021 Jun 7;118(24):e2025053118. doi: 10.1073/pnas.2025053118 (PMC8214690; doi:10.1073/pnas.2025053118)
Supplement: Supplementary File [file pnas.2025053118.sapp.pdf]

Supplementary Information for

ALS-associated missense mutations in TBK1 differentially disrupt mitophagy

Olivia Harding[1], Chantell S. Evans[1], Junqiang Ye[2,3], Jonah Cheung[4], Tom Maniatis[2,3,5], and Erika L.F. Holzbaur[1*]

*Corresponding Erika L.F. Holzbaur
holzbaur@pennmedicine.upenn.edu

MATERIALS AND METHODS (EXTENDED)

Reagents. Constructs used were: Mito-DsRed2 (kindly provided by. T. Schwarz, Harvard Medical School, Boston) and SBFP2-mito (Mito-DsRed2 recloned into pSBFP2-C1, Addgene #22880); Mito-SNAP (recloned from Mito-DsRed into a pSNAPf [New England Biolabs]), YFP-Parkin (a gift from R. Youle, NIH, Bethesda, MD) and untagged Parkin (subcloned from YFP-Parkin); pEGFP-OPTN (kindly provided from I. Dikic, Goethe University, Frankfurt), Halo-OPTN (subcloned from EGFP-OPTN to a pHaloTag vector, Promega); pEGFP-LC3B (a gift from T. Yoshimori, Osaka University, Osaka); and SNAP-tagged or Halo-tagged versions of all TBK1 variants. siRNA targeting the 5' (UAACAAGAGGAUUGCCUGA) and 3' (CCACUGUUAUACUGGGAUA) ends of hTBK1 and a scrambled control siRNA were from Horizon Discovery, and used on HeLa-M cells. ON-TARGET*Plus* Rat TBK1 (299827) siRNA *SMARTpoo*l (L-101406-02-0005; Horizon) were used on neurons. SNAP ligands (SNAP-Cell 647-SiR, S9102S and SNAP-Cell 430, S9109S) were from New England BioLabs. Halo ligands (JaneliaFluor 646, GA112A and TMR, G8252) were from Promega. TMRE (tetramethylrhodamine ethyl ester, Ethyl Ester, Perchlorate) was purchased from Life Technologies, (T-669). Antibodies used were: anti-TBK1 (abcam, ab40676, IF: 1:100, and Novus Biologicals, 108A429, WB: 1:1000), anti-SNAP (New England BioLabs, P9310, WB: 1:1000), and anti-phospho-S177-OPTN (Cell Signaling Technology, 57548, IF: 1:200). The drug carbonyl cyanide 3-chlorophenylhydrazone (CCCP) was purchased from Millipore Sigma (C2759). Antimycin A (A8674) and Oligomycin A (75351) were purchased from Sigma-Aldrich. ULK-101 (S8793) was purchased from Selleckchem.

Cell culture and transfection. HeLa-M cells and HEK293T cells were maintained in DMEM (Corning, 10-017-CV) with 10% fetal bovine serum (HyClone) and 1% GlutaMAX glucose supplement (Gibco, 35050061). Cells were maintained in an environment of 37 ºC with 5% CO_2. 48 hours prior to fixation or live imaging, 0.28 million cells were plated on each glass-bottomed 35 mm dish (MatTek, P35G-1.5-20-C). HEK cells were plated onto glass coverslips that had been pre-coated with 0.5 mg/ml poly-L-lysine (Sigma Aldrich) for 24 hr in order to prevent sloughing. 18-20 hours prior to fixation or live imaging, cells were approximately 80-90% confluent, and were transfected with the appropriate constructs using Lipofectamine 2000 (ThermoFisher Scientific, 11668027) at a 1:4 ratio of mass (ug) to volume (uL). siRNA was transfected simultaneously at 40 μM.

Primary hippocampal culture and transfection: A suspension of embryonic day 18 Sprague Dawley rat hippocampal neurons were provided from the Neurons R Us Culture Service Center at the University of Pennsylvania. Cells were plated on 35 mm glass-bottom dishes (MatTek) at a density of 250,000 cells/dish; dishes were precoated with 0.5 mg/ml poly-L-lysine (Sigma Aldrich). Cells were initially plated in MEM supplemented with 10% horse serum, 33 mM D-glucose, and 1 mM sodium pyruvate and left for 2-5 hours. The media was then replaced with Neurobasal (Gibco) supplemented with 33 mM D-glucose, 2 mM GlutaMAX (Invitrogen), 100 units/ml penicillin, 100 μg/ml streptomycin, and 2% B-27 (ThermoFisher) (Maintenance Media; MM) and cells were maintained at 37 C in a 5% CO_2 incubator. AraC (5 μM) was added the day after plating to prevent glia cell proliferation. Neurons were transfected at 5 DIV with DNA (0.8-1.2 μg of total plasmid) and siRNA (45 pmol) mixtures using Lipofectamine 2000 Transfection Reagent (ThermoFisher) and incubated 36-48 hours. To induce mitophagy, media was fully replaced with MM containing 3 nM Antimycin A for 2 hours; in control conditions media was replaced with standard MM.

Labeling, treatment, and fixation. <u>HeLa-M and HEK cells:</u> To tag Halo-tagged or SNAP-tagged proteins, cells were incubated with the respective Halo or SNAP ligands. For Halo, cells were incubated with 190 nM Halo ligand for at least 20 min. Cells were then washed two times with conditioned media and allowed to rest for at least 20 min in conditioned media. For SNAP, cells were incubated with 1.25 μM SNAP ligand for at least

1 hour. Cells were then washed two times with conditioned media and allowed to rest for at least 30 min in conditioned media. When both SNAP and Halo were used, cells were incubated with Halo tag first, then SNAP tag, with their respective protocols. Cells were washed two more times with conditioned media. When applicable, cells were treated with 5 µM ULK-101 for 2.5 hr. Cells were then treated with 20 µM CCCP or a combination of 10 µM Oligomycin A and 10 µM Antimycin A in conditioned media for 1.5 hours. Immediately afterward, cells were washed with warmed PBS then fixed with warmed 4% paraformaldehyde for 10-12 min. For experiments with antibody tagging, cells were permeabilized with 0.5% Triton X at room temperature for 5 min, then blocked with 3% BSA, 0.2% Triton X for one hour. Cells were incubated with primary antibodies overnight at 4 ºC. Afterward, cells were washed 4x 5 min in PBS and incubated with secondary antibodies for one hour. Cells were then washed 4x 5 min in PBS and imaged. For the transfection levels test (Supplemental Figure 1C), after fixation cells were labeled with Hoechst 33342 for 5 min, then again washed 4x 6 min before imaging. Hippocampal neurons: Prior to imaging, Halo-tag (JaneliaFluor 646, 100 nM) and SNAP-tag (SNAP-Cell 430, 2 µM) ligands were added for 30 min, followed by two quick washes and a 30 min washout. Mitochondrial membrane potential was assessed by loading mitochondria with 2.5 nM TMRE for 30 min.

Fixed and live cell imaging. HeLa-M and HEK293T cells: For the transfection levels test in HeLa-M cells (Supplemental Figure 1C), cells were imaged with widefield microscopy. Images were taken from three fields per dish, and the only requirement for each field was that the nuclei (Hoechst staining) appeared healthy and regularly spaced in an area that was close to fully confluent. For all other experiments, samples were imaged with a Nikon Eclipse Ti Microscope with a 100X objective (Apochromat, 1.49-N.A. oil immersion) and an UltraView Vox spinning disk confocal system (PerkinElmer). Z-stacks at 0.15 nm/step or timelapse confocal images at 30 seconds/frame were collected with Volocity acquisition software (PerkinElmer). Fields of view were chosen to maximize the number of cells that expressed detectable components of interest. In fixed samples, z-stacks were collected through the majority of cells' midsections.

For live cell imaging, conditioned media was replaced with Leibovitz's L-15 Medium (Gibco, 11415064) supplemented with 10% fetal bovine serum. Cells were then rested for at least 10 min in the 37 ºC imaging chamber of the microscope. For timelapse mitochondrial damage, a z position was chosen in the midsection of a healthy-appearing cell with a regularly shaped nucleus (nucleus characterized by absence of tagged TBK1). 5-10 frames were collected at basal conditions, then a volume of imaging media at least 50% of the initial volume was added, including CCCP to bring the total concentration to 20 µM as frame collection continued.

Hippocampal neurons: Neurons were imaged in HibernateE (Brain Bits) supplemented with 2% B27 and 33 mM D-glucose; Antimycin A was added to the imaging media for treated conditions. TMRE was added to the imaging media for TMRE experiments.

Mitochondrial enrichments and immunoblots. For standard cell lysis, cells were washed two times with warmed PBS, then lysed with ice cold RIPA buffer (50 mM Tris-HCl, 1 mM EDTA, 2 mM EGTA, 1% Triton X, 0.5% sodium deoxycholate, 0.1% sodium dodecyl sulfate, 150 mM NaCl) with added Halt Protease and Phosphatase Inhibitor Cocktail (ThermoFisher Scientific, 78444) and scraped into sample tubes and incubated with continuous gentle inversion at 4 ºC for 20 min. Samples were then spun at 4 ºC in a microcentrifuge at 17 g for 20 min, and the supernatant was transferred to a separate tube. Samples were assayed for protein concentration with Pierce BCA Protein Assay Kit (ThermoFisher Scientific, 23225). Mitochondrial enrichment was performed with ThermoScientific isolation kit for cultured cells (89874) and mitochondrial fractions were diluted in RIPA buffer with Halt Cocktail as above.

30 µg of each sample was loaded into a 10% gel (or 14% gel for TOM20 detection). After electrophoresis, protein bands were transferred to PVDF membrane (Immobilon-FL, Millipore Sigma, IPFL00010) and total protein was labeled with Revert 700 (Li-Cor, 926-11011) and imaged on an Odyssey CLx machine (Li-Cor). After clearing the total protein stain with a solution of 0.1% NaOH, 30% methanol, the membrane was blocked with TrueBlack blocking solution (Biotium, 23013-T) for 1 hour at room temperature. Primary antibodies were incubated overnight at 4 ºC in TrueBlack WB antibody diluent (Biotium,

#23013). After primary antibody incubation, the membrane was washed 4x 5 min in TBS with 0.2% TWEEN-20. Secondary fluorescent antibodies (Li-Cor IRDyes, 926-68073, 926-32212) were diluted at 1:20,000 for 1 hour at room temperature in TrueBlack WB diluent. Finally, the membrane was washed 4x 5 min again with TBST before it was imaged again.

Image processing and analysis. Images were deconvolved with Huygen's Professional version 17.10 software (Scientific Volume Imaging, The Netherlands, http://svi.nl) to remove background noise and increase resolution and signal-to noise ratio. The Classic Maximum Likelihood Estimation (CMLE) algorithm with theoretical PSF was performed for 50 iterations at most. The signal-to-noise ratio for all channels was set between 10 and 30, depending on the individual construct; all other settings were default. Images were assembled in Illustrator (Adobe). Most images shown are deconvolved, with the exceptions of widefield images (Supplemental Figure 1C), all Supplemental whole-field images, the experiment to determine ULK1 dependence (Figure 6), and neuronal mitochondrial TMRE images (Figure 8A). All intensity measurements were carried out on raw intensity images, not deconvolved images.

In Figures 1E,F, 2, 3, 5, and 6, maximal projections of 3.75 μm were generated through the central volume of the cells. For other analyses, confocal sections were used. Parkin, OPTN, TBK1, and LC3 rings were delineated by hand and measured in ImageJ (57). Patterns of OPTN and TBK1 were classified as rings if they coincided with Parkin signal around a rounded mitochondrion. At least half of the ring had to be clearly evident to be counted. Diameters and intensities were measured for all ring ROIs in each cell and averaged. Thus, each point is the average for a single cell. To quantify percentage of LC3-positive mitochondria, Ilastik software version 1.2.2 (58) was trained to categorize mitochondria using the dsRed2-mitochondria channel in the Pixel Classification module. Feature selection used color/intensity, edge, and texture up to $\sigma = 5$ pixels. Binary images were exported as .tifs using simple segmentation, and the Analyze Particles function of ImageJ was used to count mitochondria. Mitochondria were considered positive for LC3 if the fluorescent LC3 ring surrounded at least half of the organelle. Each data point represents the percentage of LC3-positive mitochondria for a single cell. For Figure 5, OPTN rings were identified as before. Then OPTN ring regions of interest (ROIs) were

transferred to the TBK1 and phospho-OPTN channels of the same image, and mean fluorescence intensity was measured for each ROI on the non-deconvolved images. Intensity data points were plotted for WT-TBK1-expressing cells, and all intensities above the 25th percentile were considered “positive,” while all intensities below were considered “negative” (see Supplemental Figure 6B,C). Thus, every OPTN ring could be categorized as positive or negative for phospho-OPTN and TBK1. Images were blinded before ring counting. For ULK1 experiments, outlines were drawn around whole cells and the average intensity in the region was measured in FIJI.

For timelapse imaging, events were quantified if they remained within the z frame for most of the sequence. Background was calculated by measuring mean intensity in the JF696 channel of a ROI drawn in an area of the cell with no detectable events. This mean was subtracted from the ROI for the event at each point over the timelapse. Hence, the M559R-TBK1 signal falls below zero, since there is some photo-bleaching of the signal over time.

For volume rendering, deconvolved .tif files were converted to .ims files with the Imaris File Converter and processed with Imaris software (Oxford Instruments). Normal shading mode was used to render images of the respective volumes of mitochondria and mitophagy components of representative events.

For hippocampal neurons, following image processing, protein ring formation and mitochondrial fragments were manually identified and quantified using Fiji, where only clearly defined structures were quantified. Fluorescence intensities (TMRE and Halo-TBK1) were quantified from max projections of unprocessed z-stack images in Fiji. The values of five individual areas (2.2 x 2.2 μm square) in the soma were averaged to determine the mean gray value for each cell. Mitochondrial content was determined by dividing the mitochondrial volume by the somal volume for each cell, where volume measurements were determined using the volume measurement function in Volocity Quantitation (Quorum Technologies). The mitochondrial aspect ratio was determined using the Ridge Detection Plugin with the SLOPE method for overlap resolution on single plane images. The Particle Analyzer tool with Shape Descriptors and the Aspect Ratio (AR) function was used to quantify the mitochondrial aspect ratio for individual

mitochondria within the neuron. Prism (GraphPad) was used to plot all graphs and determine statistical significance. Adobe Illustrator was used to prepare all figures and images.

Transfection level images included Hoechst and JF646 channels (Supplemental Figure 1C). Both channels were maximally projected, and the JF696 channel was background subtracted with ImageJ's rolling ball radius set to 25.0 pixels, with sliding paraboloid and disabled smoothing. Images were then imported to CellProfiler software (59) and nuclei were identified as primary objects; then cells were delineated by the propagation method in the JF646 channel. Thus, only cells expressing SNAP-TBK1 were identified and the mean intensities of their cytoplasmic signals were exported to Excel. These values were displayed on a histogram to demonstrate the relative frequencies of mean intensities (GraphPad).

For immunoblots, ImageStudio Software (Version 5, Li-Cor) was used to scan bands to ensure no patches were overexposed. ImageStudio was used to subtract background and quantify band intensities, which were normalized to the total protein signal for their respective lanes with Excel (Microsoft). For mitochondrial enrichment, bands were normalized to TOM20. Those values were graphed in GraphPad (Version 9, Prism).

SUPPLEMENTAL FIGURES

SUPPLEMENTAL FIGURES

Supplemental Figure 1

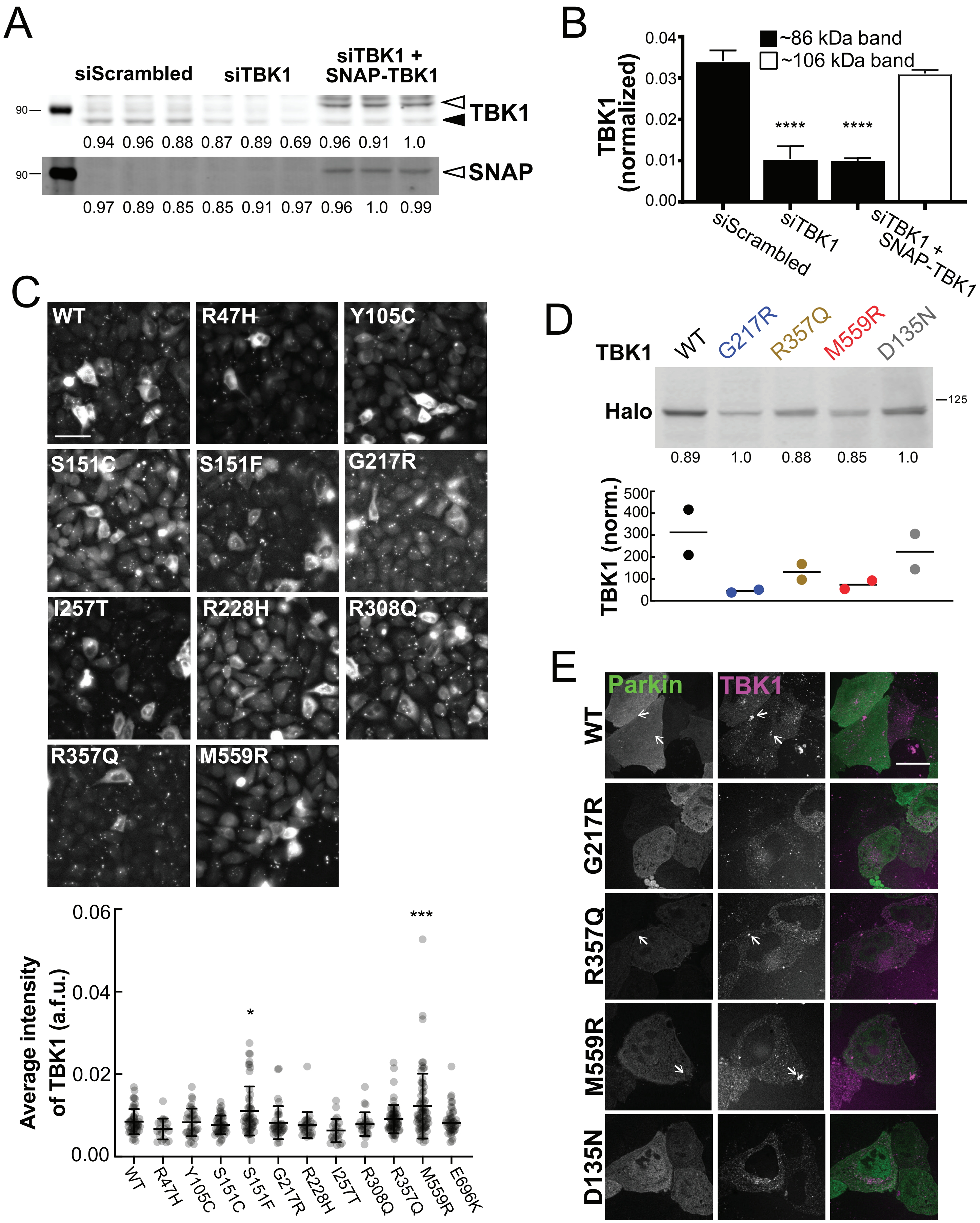

Supplemental Figure 1. TBK1 is depleted and tagged TBK1 is exogenously expressed in a HeLa-M system.

A. Western blot of HeLa cells under mock, knockdown, and rescue conditions. Samples were probed for total TBK1 (top panel) and for SNAP (bottom panel). TBK1 is approximately 86 kDa (solid black arrowhead, top panel), and SNAP-TBK1 is expected to appear at 106 kDa (white arrowheads, both panels). Numbers to the left of blots indicate kDa. B. Band intensities were quantified and normalized to total protein, as indicated by the numbers below each lane in (A). Error bars indicate SEM. n= 3 independent collections. **** $p < 0.0001$ by ordinary one-way ANOVA with Dunnett's multiple comparisons test. C. Representative widefield images of fixed HeLa cells in basal conditions, depleted of endogenous TBK1 and expressing siRNA-resistant SNAP-tagged TBK1 variants (grayscale). Scale bar, 60 µm. Below, graph of TBK1 average signal intensities for cells transfected with the respective TBK1 constructs. Bars indicate mean with SD. * $p \leq 0.05$, *** $p < 0.001$ by ordinary one-way ANOVA with Dunnett's multiple comparisons test. D. Representative Western blot of HeLa cell lysates depleted of endogenous TBK1 and expressing the respective Halo-tagged TBK1 variants (~119 kDa). Quantification of Halo band relative to total protein shown below (n = 2). Number to the left of blot indicates kDa. E. Representative confocal images of fixed HeLa cells in basal conditions, depleted of endogenous TBK1 and expressing Parkin (green) and siRNA-resistant Halo-tagged TBK1 variants (magenta). Arrows indicate TBK1 aggregates. Scale bar, 20 µm.

Supplemental Figure 2

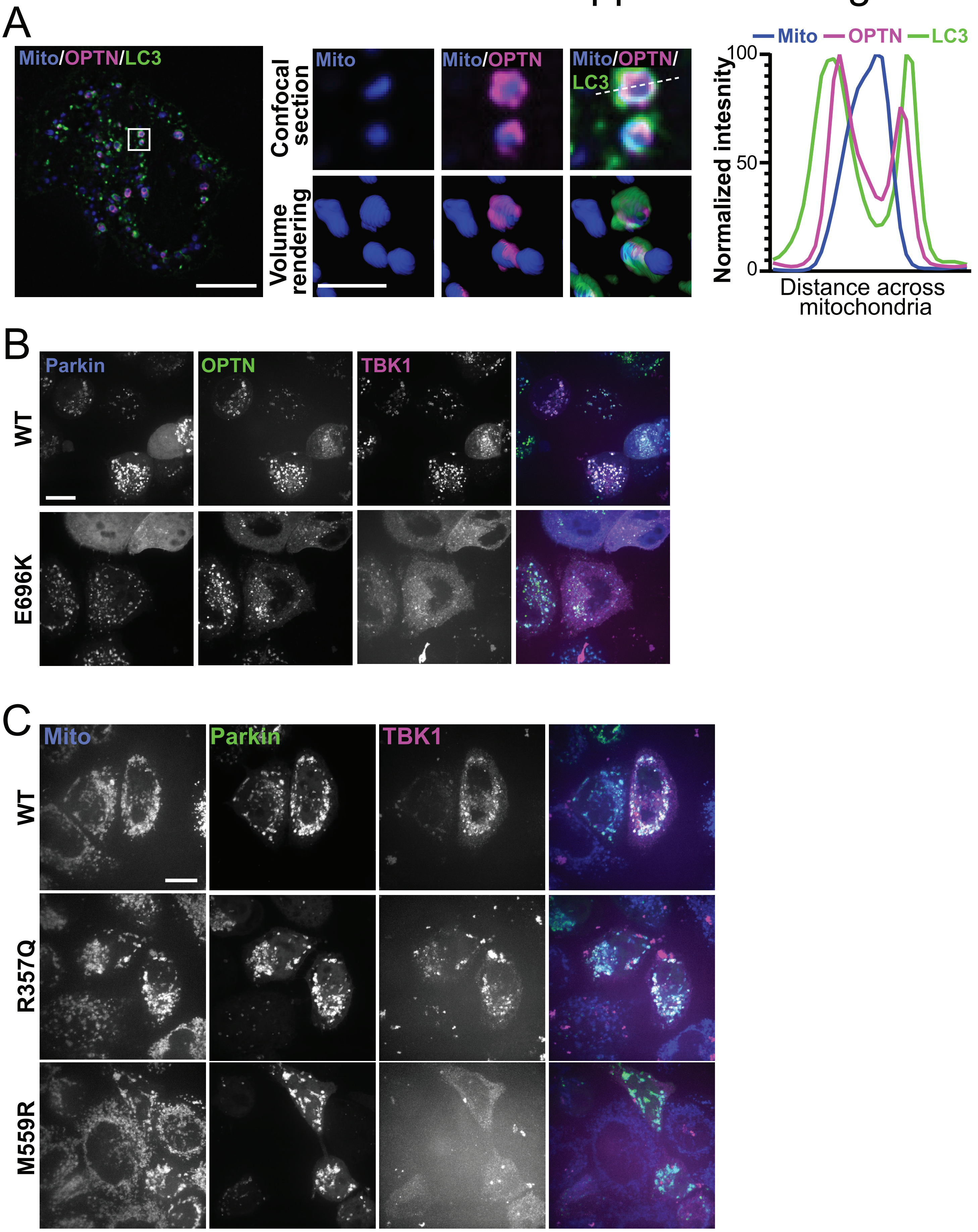

Supplemental Figure 2. OPTN and LC3 are recruited to damaged mitochondria, and corresponding whole-field images for Main Figures 1,2.

A. Confocal section of a HeLa cell expressing Parkin (not tagged), a mitochondrial maker (blue), OPTN (magenta) and LC3 (green), fixed after treatment with CCCP for 90 min. The inset (white box) and zoom images (right, top row) exhibit two mitochondria that have recruited OPTN and LC3. A volume rendering is shown below (right, bottom row). Scale bars, zoom out, 10 μm; zoom in, 2 μm. Right, profiles of relative signal intensities for mitochondria (blue line), OPTN (magenta line), and LC3 (green line) are quantified across the diameter of the rounded mitochondria (white dashed line in zoom image). B. Representative whole-field images corresponding to images in Main Figure 1E,F. Scale bar, 25 μm. C. Representative whole-field images corresponding to images in Main Figure 2A. Scale bar, 25 μm.

Supplemental Figure 3

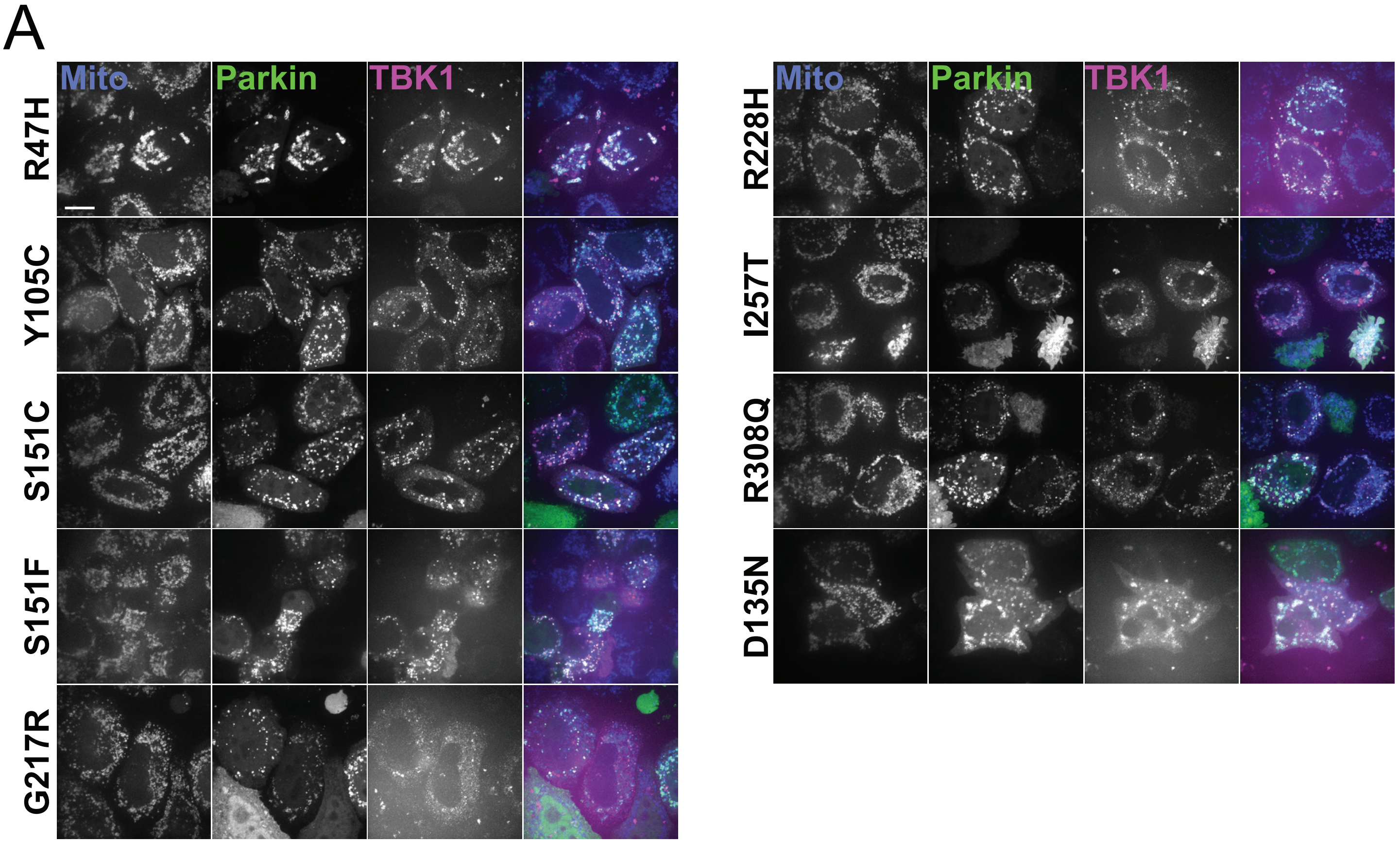

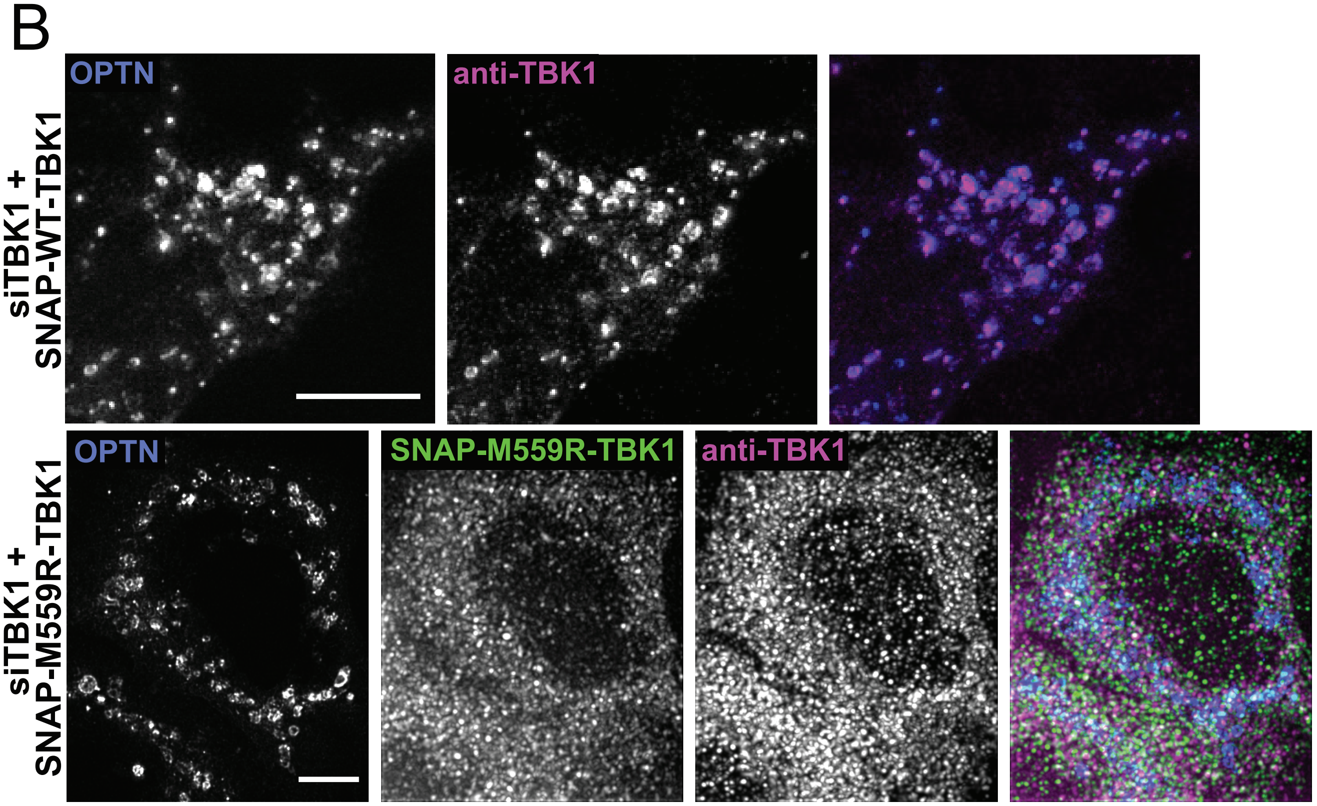

Supplemental Figure 3. Corresponding whole-field images for Main Figure 3, and comparison of total TBK1 recruitment to damaged mitochondria in WT- versus M559R-TBK1 expressing cells.

A. Representative whole-field images corresponding to images in Main Figure 3A. Scale bar, 25 μm. B. Maximum intensity projection image of HeLa cells depleted of endogenous TBK1 and expressing Halo-OPTN (blue) and WT-TBK1 (top row, not labeled) or M559R-TBK1 (bottom row, green), fixed after 90 min treatment with CCCP. Cells were tagged with an antibody to total TBK1 (magenta). Scale bars, 10 μm.

Supplemental Figure 4

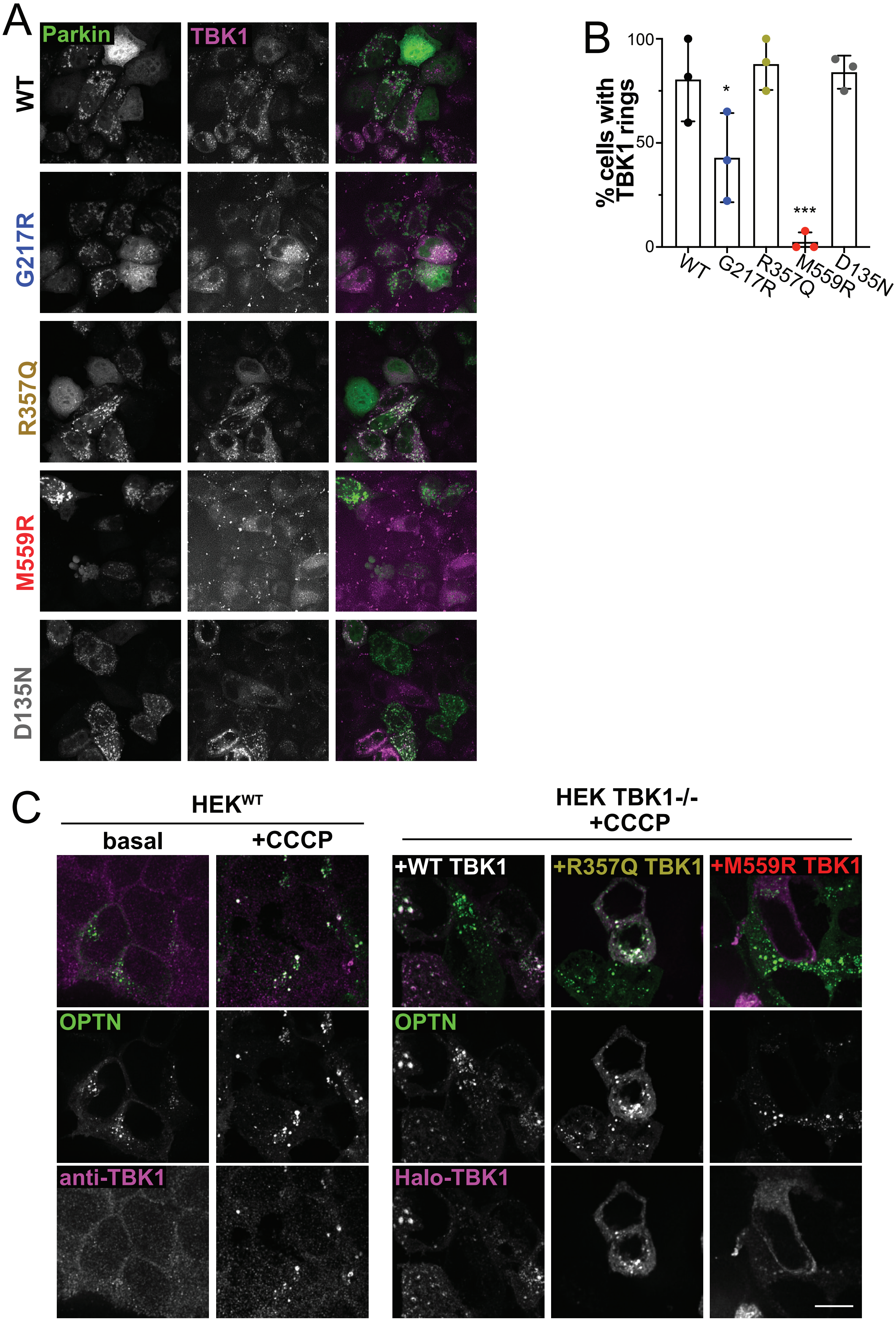

Supplemental Figure 4. Treatment with Antimycin A and Oligomycin A induces mitochondrial depolarization and TBK1 recruitment to damaged mitochondria, and HEK cell mitochondria recruit OPTN and TBK1.

A. Maximum intensity projection image of HeLa cells depleted of endogenous TBK1, expressing Parkin (green) and the respective TBK1 mutants (magenta), fixed after 90 min treatment with Antimycin A/Oligomycin A. B. Quantification of percentages of cells observed that exhibit clear TBK1 rings that coincide with Parkin. * $p \leq 0.05$, *** $p < 0.001$ by ordinary one-way ANOVA with Dunnett's multiple comparisons test. Error bars indicate SD. n= 3 independent experiments. C. WT or TBK1-/- HEK cells expressing EGFP-OPTN (green) and fixed. WT HEK cells were tagged with an antibody to TBK1 (magenta) in basal conditions or after 90 min CCCP treatment. TBK1-/- HEK cells expressed WT-, R357Q-, or M559R-TBK1 (magenta) before treatment with CCCP and fixation. Scale bar, 10 µm.

Supplemental Figure 5

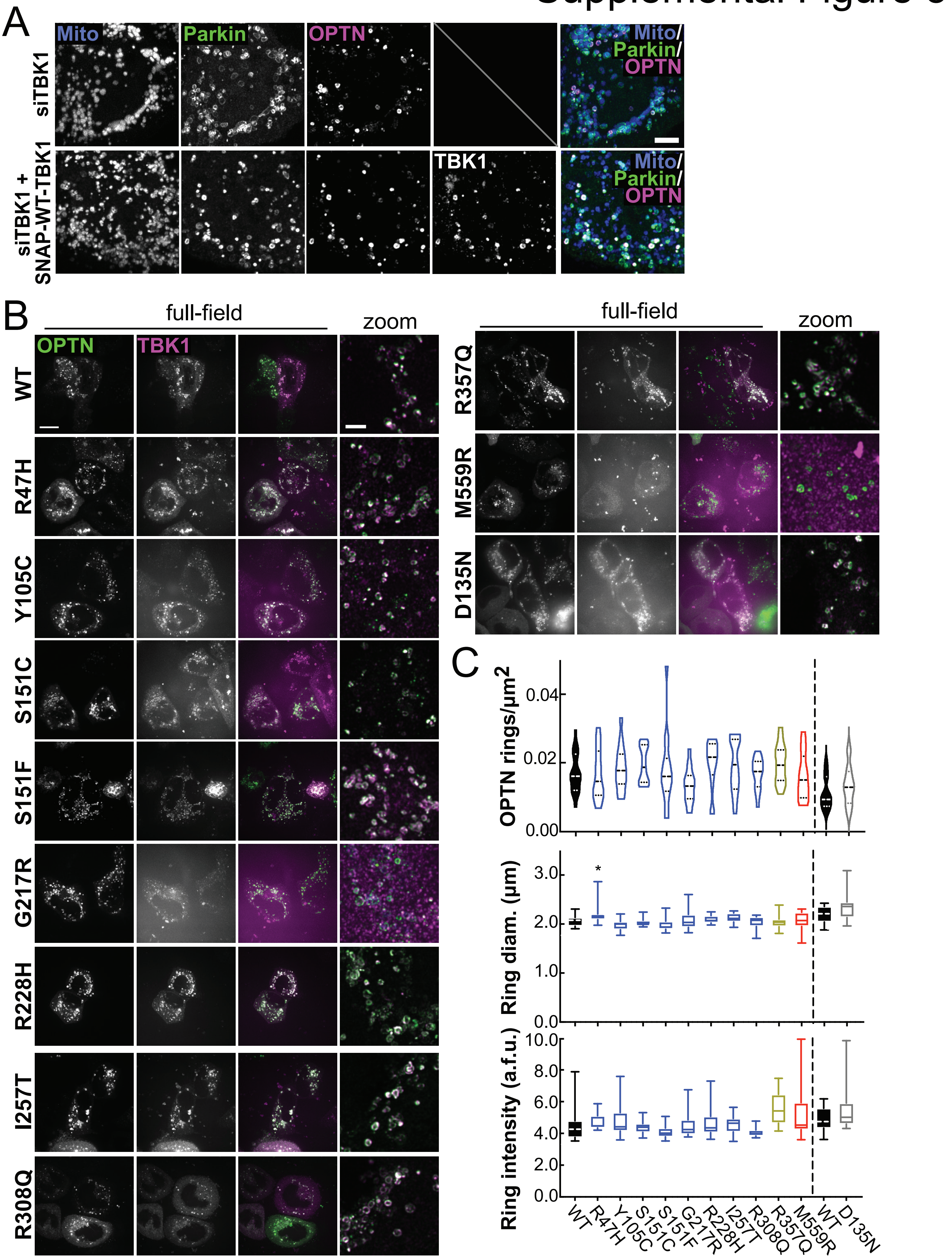

Supplemental Figure 5. OPTN is recruited to damaged mitochondria despite depletion of endogenous TBK1.

A. Maximum intensity projection images of fixed HeLa cells depleted of endogenous TBK1, expressing a mitochondrial-localized fluorophore (blue), Parkin (green), and OPTN (magenta), fixed after 90 min treatment with CCCP. In the bottom panels, cells were rescued with exogenous SNAP-WT-TBK1, displayed in grayscale in the bottom right panel (TBK1 not included in the merged image). Scale bar, 10 µm. B. HeLa cells depleted of endogenous TBK1 and expressing Parkin, OPTN. (green), and TBK1 variants (magenta), fixed after treatment with CCCP for 90 min. First three columns are whole-field view. Scale bar, 10 µm. Final column is zoom of merged channels. Scale bar, 5 µm. C. Quantification of OPTN rings/μm^2, ring diameter (diam.), and ring signal intensity. For ring density, horizontal dashed lines, median; horizontal dotted lines, 25th and 75th quartiles. Vertical dashed lines distinguish separate data sets. n= 14-20 cells from at least 3 independent experiments. * $p \leq 0.05$ by ordinary one-way ANOVA with Dunnett's multiple comparisons test. Arbitrary fluorescent units, a.f.u.

Supplemental Figure 6

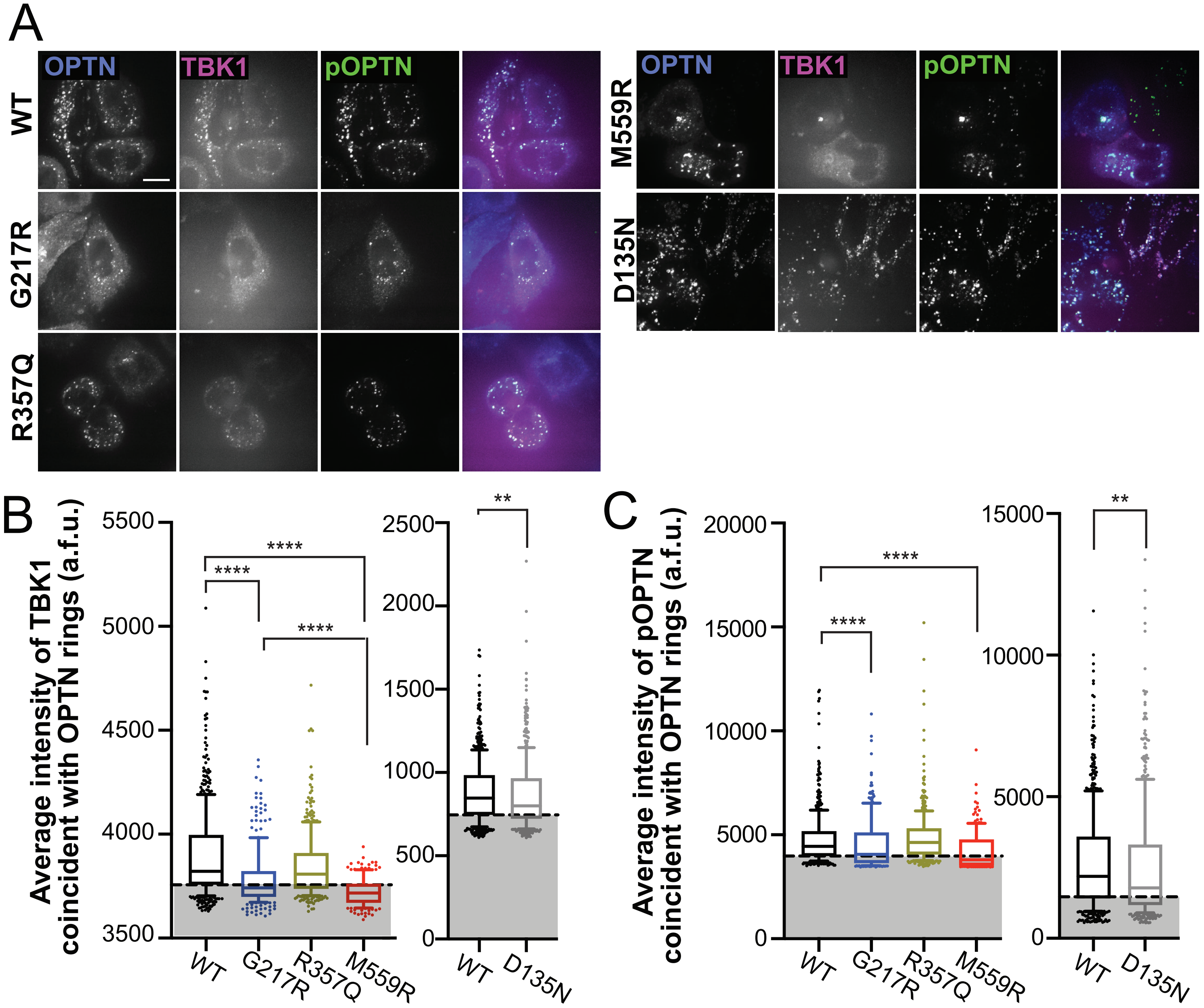

Supplemental Figure 6. Corresponding whole-field images for Main Figure 6, and raw intensities of TBK1 and phospho-OPTN signals with different TBK1 mutants expressed.

A. Representative whole-field images corresponding to images in Main Figure 6A-E. Scale bar, 25 μm. B,C. Raw TBK1 (A) and phospho-OPTN (B) intensity measurements for OPTN rings in the respective TBK1 variant-expressing cells. Black dashed horizontal lines indicate 25th percentile cutoff. Graphs are divided into experiments carried out more than one year apart. Statistical analysis among graphs of four mutant expressions (WT, G217R, R357Q, and M559R) were carried out with Kruskal-Wallis test with Dunn's multiple comparisons. For analysis between two mutant expressions (WT and D135N), Mann-Whitney test was used. ** $p < 0.001$, **** $p < 0.0001$ Arbitrary fluorescent units, a.f.u.

Supplemental Figure 7

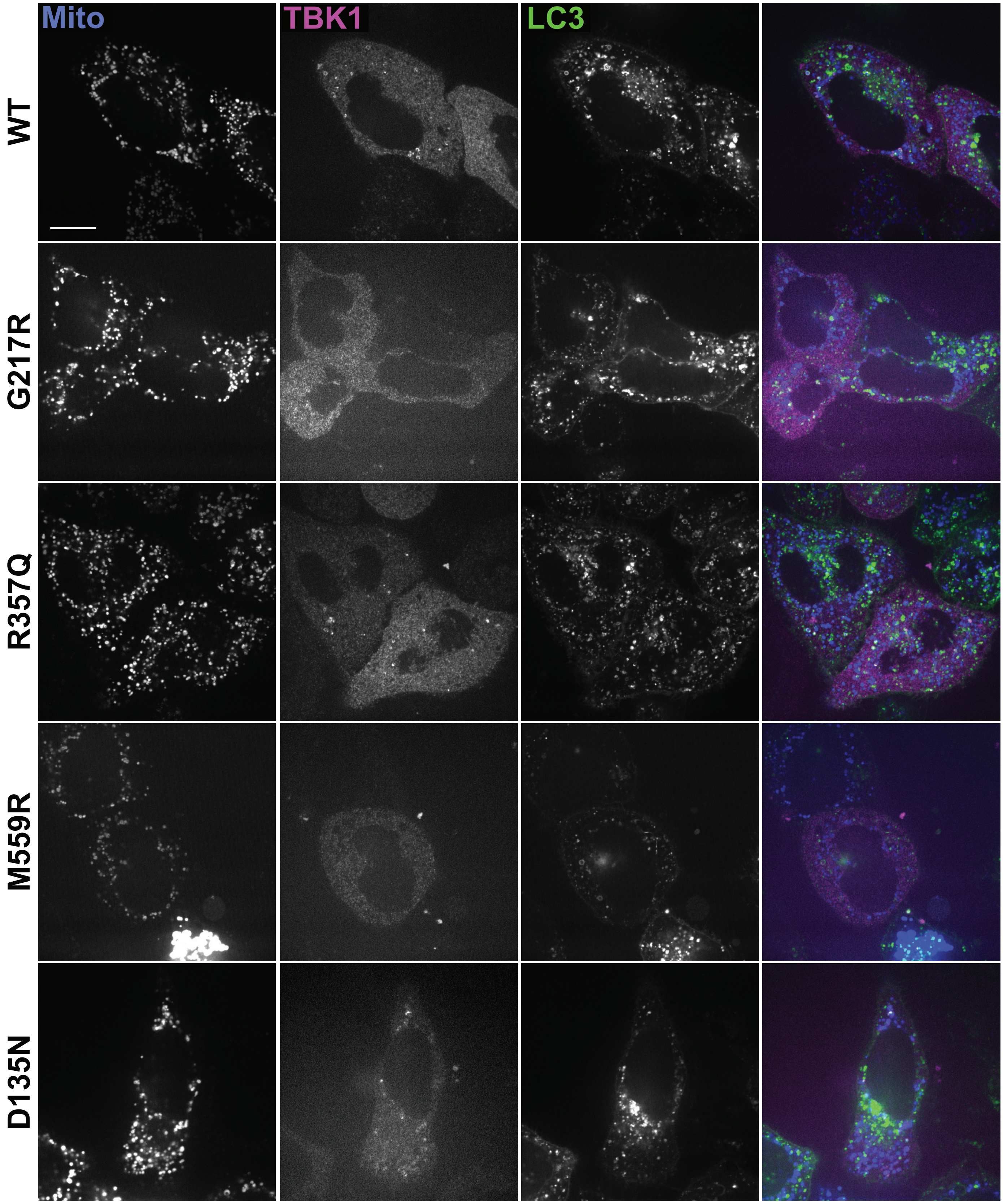

Supplemental Figure 7. Representative whole-field images corresponding to images in Main Figure 7.

Scale bar, 25 μm.

Supplemental Figure 8

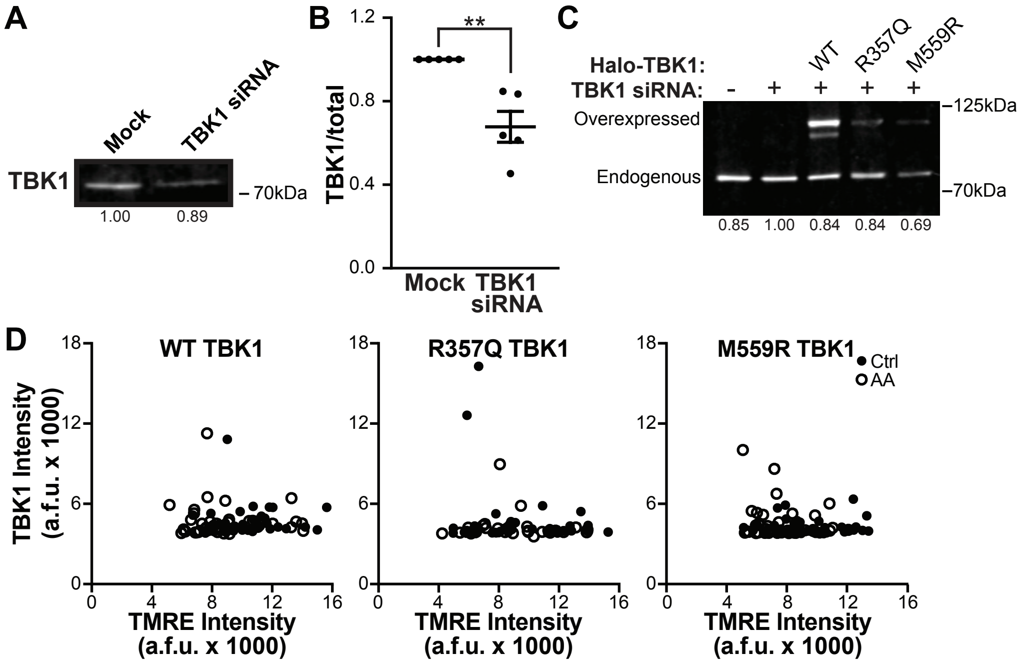

Supplemental Figure 8. TBK1 is efficiently knocked down in neurons with siRNA.

A,B. Representative Western blot (A) and quantification (B) of neurons after treatment with mock or TBK1 siRNA. Data shown as the fold change over control of TBK1 divided by total protein stain. Normalization factors are shown under lanes. Mean $\pm$ SEM; n= 5; 7 DIV. ** $p < 0.01$ by unpaired t test. C. Western blot of non-transfected, TBK1 siRNA treated, and TBK1 siRNA treated neurons overexpressing WT or mutant Halo-TBK1. Normalization factors are shown under lanes. D. TBK1 fluorescence intensity plotted as a function of the TMRE fluorescence intensity for each cell (data also presented Figure 8B). n= 30-42 neurons from 3-4 biological replicates; 7 DIV.
